# Supplementary material for: Shotgun ion mobility mass spectrometry sequencing of heparan sulfate saccharides
Source: Nat Commun. 2020 Mar 20;11:1481. doi: 10.1038/s41467-020-15284-y (PMC7083916; doi:10.1038/s41467-020-15284-y)
Supplement: Supplementary file 1 — Supplementary Information [file 41467_2020_15284_MOESM1_ESM.pdf]

Shotgun Ion Mobility Mass Spectrometry Sequencing of Heparan Sulfate Saccharides

Rebecca L. Miller[1,2,3]*, Scott E. Guimond[2,4], Ralf Schwörer[5], Olga V. Zubkova[5], Peter. C. Tyler[5], Yongmei Xu[6], Jian Liu[6], Pradeep Chopra[7], Geert-Jan Boons[7,8], Márkó Grabarics[9,10], Christian Manz[9,10], Johanna Hofmann[9,10], Niclas G. Karlsson[11], Jeremy E. Turnbull[1,2], Weston B. Struwe[12], and Kevin Pagel[9,10].

[1]Copenhagen Center for Glycomics, Department of Cellular & Molecular Medicine, University of Copenhagen, Copenhagen N 2200, Denmark.
[2]Centre for Glycobiology, Department of Biochemistry, Institute of Integrative Biology, University of Liverpool, Crown Street, Liverpool, L69 7ZB, United Kingdom.
[3]Laboratory of Cancer Biology, Department of Oncology, Medical Sciences Division, University of Oxford, Old Road Campus Research Building, Old Road Campus, Roosevelt Drive, Oxford OX3 7DQ, United Kingdom.
[4]Institute for Science and Technology in Medicine, School of Medicine, Keele University, Keele, Staffordshire, ST5 5BG, United Kingdom.
[5]Ferrier Research Institute, Victoria University of Wellington, 69 Gracefield Road, Gracefield, Lower Hutt 5010, New Zealand.
[6]Division of Chemical Biology and Medicinal Chemistry, Eshelman School of Pharmacy, University of North Carolina, Chapel Hill, North Carolina 27599, USA.
[7]Complex Carbohydrate Research Center, University of Georgia, 315 Riverbend Road, Athens, GA 30602, USA.
[8]Department of Chemical Biology and Drug Discovery, Utrecht Institute for Pharmaceutical Science, and Bijvoet Center for Biomolecular Research, Utrecht University, Universiteitsweg 99, 3584 CG Utrecht, The Netherlands.
[9]Freie Universitaet Berlin, Institute of Chemistry and Biochemistry, Takustrasse 3, 14195 Berlin, Germany.
[10]Fritz Haber Institute of the Max Planck Society, Faradayweg 4-6, 14195 Berlin, Germany.
[11]Department of Medical Biochemistry and Cell Biology, University of Gothenburg, Box 440, Medicinaregatan 9A, 405 30 Gothenburg, Sweden.
[12]Department of Chemistry, Chemistry Research Laboratory, University of Oxford, Oxford, OX1 3QZ, United Kingdom.

Supplementary Information

Supplementary Fig. 1. SIMMS^2 method for sequencing a hexasaccharide.
Supplementary Fig. 2 SIMMS^2 sequencing of isomeric 3*O*- and 6*O*-sulfated structures
Supplementary Fig. 3. HS hexasaccharide purification and disaccharide analysis.
Supplementary Fig. 4. Purification of HS oligosaccharides with FGF1/2 bioactivity.
Supplementary Fig. 5. CCS of standard #10 using different DTIMS source conditions
Supplementary Table 1. Summary of the used library of standards.
Supplementary Table 2. Summary of CCS values obtained from the standard library.
Supplementary Table 3. CCS of B, Y, C, Z ions - tetrasaccharides UA-GlcNAc6S-UA-GlcNAc6S-R_1
Supplementary Table 4. CCS of B, Y, C, Z ions - tetrasaccharides UA-GlcNS6S-UA-GlcNS6S-R_1
Supplementary Table 5. CCS of B, Y, C, Z ions - tetrasaccharide G-GlcNS6S-I2S-GlcNS6S-R_1
Supplementary Table 6. CCS of B, Y, C, Z ions - hexasaccharide G-GlcNS6S-I-GlcNS6S-G-GlcNS6S-R_1.
Supplementary Table 7. CCS of B, Y, C, Z ions - hexasaccharide GlcNAc6S-[G-GlcNAc6S]$_2$-G-R_2.

Supplementary Table 8. CCS of B, Y, C, Z ions - hexasaccharide GlcNAc6S-[I-GlcNAc6S]$_2$-I-R_2.
Supplementary Table 9. CCS of B, Y, C, Z ions - octasaccharide GlcNAc6S-[I-GlcNAc6S]$_3$-I-R_2.
Supplementary Table 10. CCS of B, Y, C, Z ions - octasaccharide GlcNAc6S-[G-GlcNAc6S]$_3$-G-R_2.
Supplementary Table 11. CCS of B, Y, C, Z ions - octasaccharide GlcNAc6S-G-GlcNac6S-G-GlcNAc6S-I-GlcNAc6S-I-R_2.
Supplementary Table 12. CCS of B, Y, C, Z ions - octasaccharide GlcNAc6S-G-GlcNac6S-I-GlcNAc6S-I-GlcNAc6S-G-R_2.
Supplementary Table 13. CCS of B, Y, C, Z ions - octasaccharide GlcNAc6S-I-GlcNAc6S-G-GlcNAc6S-I-GlcNAc6S-G-R_2.
Supplementary Table 14. CCS of B, Y, C, Z ions - octasaccharide GlcNAc6S-G-GlcNAc6S-I-GlcNAc6S-G-GlcNAc6S-I-R_2.
Supplementary Table 15. CCS of B, Y, C, Z ions - nonasaccharide G-GlcNS-G-GlcNS-I-GlcNS-G-GlcNS-G-R_3.
Supplementary Table 16. CCS of B, Y, C, Z ions - nonasaccharide G-GlcNS-G-GlcNS-I2S-GlcNS-G-GlcNS-G-R_3.
Supplementary Table 17. CCS of B, Y, C, Z ions - decasaccharide GlcNAc6S-[I-GlcNAc6S]$_4$-I-R_2.
Supplementary Table 18. CCS of B, Y, C, Z ions - decasaccharide GlcNAc6S-[G-GlcNAc6S]$_4$-G-R_2.
Supplementary Table 19 CCS of B, Y, C, Z ions - disaccharide ΔUA2S-GlcNS3S6S
Supplementary Table 20 CCS of B, Y, C, Z ions - tetrasaccharide ΔUA-GlcNS-I2S-GlcNS3S
Supplementary Table 21 CCS of B, Y, C, Z ions - tetrasaccharide ΔUA-GlcNS6S-G-GlcNS3S6S
Supplementary Table 22 CCS of B, Y, C, Z ions - tetrasaccharide ΔUA-GlcNAc6S-G-GlcNS3S6S
Supplementary Table 23 CCS of B, Y, C, Z ions - G-GlcNS6S-G-GlcNS6S-I2S-GlcNS6S-R_1
Supplementary Table 24 CCS of B, Y, C, Z ions - G-GlcNS6S-G-GlcNS3S-I2S-GlcNS6S-R_1
Supplementary Table 25 CCS of B, Y, C, Z ions - G-GlcNS6S-G-GlcNS6S3S-I2S-GlcNS6S-R_1
Supplementary Table 26. CCS of B, Y, C, Z ions - septasaccharide ΔUA-GlcNS-I2S-GlcNS-G-GlcNS-G-R_3.
Supplementary Table 27 CCS of B, Y, C, Z -SO_3 ions - tetrasaccharides UA-GlcNAc6S-UA-GlcNAc6S-R_1
Supplementary Table 28. CCS of B, Y, C, Z -SO_3 ions - tetrasaccharides G-GlcNS6S-I-GlcNS6S-R_1
Supplementary Table 29. CCS of B, Y, C, Z -SO_3 ions – tetrasaccharides I-GlcNS6S-G-GlcNS6S-R_1
Supplementary Table 30. CCS of B, Y, C, Z -SO_3 ions - tetrasaccharide G-GlcNS6S-I2S-GlcNS6S-R_1
Supplementary Table 31. CCS of B, Y, C, Z -SO_3 ions - hexasaccharide G-GlcNS6S-I-GlcNS6S-G-GlcNS6S-R_1.
Supplementary Table 32. CCS of B, Y, C, Z -SO_3 ions - hexasaccharide GlcNAc6S-[G-GlcNAc6S]$_2$-G-R_2.
Supplementary Table 33. CCS of B, Y, C, Z -SO_3 ions - hexasaccharide GlcNAc6S-[I-GlcNAc6S]$_2$-I-R_2.
Supplementary Table 34 CCS of B, Y, C, Z -SO_3 ions - GlcNAc6S-[I-GlcNAc6S]$_3$-I-R_2
Supplementary Table 35. CCS of B, Y, C, Z -SO_3 ions - octasaccharide GlcNAc6S-[G-GlcNAc6S]$_3$-G-R_2.
Supplementary Table 36. CCS of B, Y, C, Z -SO_3 ions - octasaccharide GlcNAc6S-G-GlcNAc6S-G-GlcNAc6S-I-GlcNAc6S-I-R_2.
Supplementary Table 37. CCS of B, Y, C, Z -SO_3 ions - octasaccharide GlcNAc6S-G-GlcNAc6S-I-GlcNAc6S-I-GlcNAc6S-G-R_2.
Supplementary Table 38. CCS of B, Y, C, Z -SO_3 ions - octasaccharide GlcNAc6S-I-GlcNAc6S-G-GlcNAc6S-I-GlcNAc6S-G-R_2.
Supplementary Table 39. CCS of B, Y, C, Z -SO_3 ions - octasaccharide GlcNAc6S-G-GlcNAc6S-I-GlcNAc6S-G-GlcNAc6S-I-R_2.
Supplementary Table 40. CCS of B, Y, C, Z -SO_3 ions - nonasaccharide G-GlcNS-G-GlcNS-I-GlcNS-G-GlcNS-G-R_3.
Supplementary Table 41. CCS of B, Y, C, Z -SO_3 ions - nonasaccharide G-GlcNS-G-GlcNS-I2S-GlcNS-G-GlcNS-G-R_3.
Supplementary Table 42. CCS of B, Y, C, Z -SO_3 ions - decasaccharide GlcNAc6S-[I-GlcNAc6S]$_4$-I-R_2.
Supplementary Table 43. CCS of B, Y, C, Z -SO_3 ions - decasaccharide GlcNAc6S-[G-GlcNAc6S]$_4$-G-R_2.
Supplementary Table 44 CCS of B, Y, C, Z -SO_3 ions - disaccharide ΔUA2S-GlcNS3S6S
Supplementary Table 45 CCS of B, Y, C, Z -SO_3 ions - tetrasaccharide ΔUA-GlcNS-I2S-GlcNS3S

Supplementary Table 46 CCS of B, Y, C, Z -SO_3 ions - tetrasaccharide ΔUA-GlcNS6S-G-GlcNS3S6S
Supplementary Table 47 CCS of B, Y, C, Z -SO_3 ions - tetrasaccharide ΔUA-GlcNAc6S-G-GlcNS3S6S
Supplementary Table 48 CCS of B, Y, C, Z ions - G-GlcNS6S-G-GlcNS6S-I2S-GlcNS6S-R_1
Supplementary Table 49 CCS of B, Y, C, Z ions - G-GlcNS6S-G-GlcNS3S-I2S-GlcNS6S-R_1
Supplementary Table 50 CCS of B, Y, C, Z ions - G-GlcNS6S-G-GlcNS6S3S-I2S-GlcNS6S-R_1
Supplementary Table 51. CCS of B, Y, C, Z -SO_3 ions - septasaccharide ΔUA-GlcNS-I2S-GlcNS-G-GlcNS-G-R_3.
Supplementary Table 52. Theoretically ions - dp6+3SO_3+1NAc, ΔUA-GlcNS-UA-GlcNAc-UA2S-GlcNS.
Supplementary Table 53 Theoretically ions - dp6+3SO_3+1NAc, ΔUA-GlcNS-UA2S-GlcNS-UA-GlcNAc.
Supplementary Table 54. Theoretically ions - dp6+3SO_3+1NAc, ΔUA-GlcNAc-UA-GlcNS-UA2S-GlcNS
Supplementary Table 55. Theoretically ions - dp6+3SO_3+1NAc, ΔUA-GlcNAc-UA2S-GlcNS-UA-GlcNS.
Supplementary Table 56. Theoretically ions - dp6+3SO_3+1NAc, ΔUA2S-GlcNS-UA-GlcNS-UA-GlcNAc.
Supplementary Table 57. Theoretically ions - dp6+3SO_3+1NAc, ΔUA2S-GlcNS-UA-GlcNAc-UA-GlcNS.
Supplementary Table 58. Summary overview of the B, Y, C and Z ions for dp6+3SO_3+1NAc.
Supplementary Table 59. CCS of B, Y, C and Z ions - ΔUA-GlcNS-I2S-GlcNS-G-GlcNAc.
Supplementary Table 60 CCS of B, Y, C and Z -SO_3 ions - ΔUA-GlcNS-I2S-GlcNS-G-GlcNAc
Supplementary Table 61. Theoretically ions - dp6+5SO_3+1NAc, ΔUA-GlcNS-UA-GlcNAc6S-UA2S-GlcNS6S.
Supplementary Table 62. Theoretically ions - dp6+5SO_3+1NAc, ΔUA-GlcNS-UA2S-GlcNS6S-UA-GlcNAc6S.
Supplementary Table 63. Theoretically ions - dp6+5SO_3+1NAc, ΔUA-GlcNAc6S-UA-GlcNS-UA2S-GlcNS6S
Supplementary Table 64. Theoretically ions - dp6+5SO_3+1NAc, ΔUA-GlcNAc6S-UA2S-GlcNS6S-UA-GlcNS
Supplementary Table 65. Theoretically ions - dp6+5SO_3+1NAc, ΔUA2S-GlcNS6S-UA-GlcNS-UA-GlcNAc6S
Supplementary Table 66. Theoretically ions - dp6+5SO_3+1NAc, ΔUA2S-GlcNS6S-UA-GlcNAc6S-UA-GlcNS
Supplementary Table 67. Summary overview of the B, Y, C and Z ions for dp6+5SO_3+1NAc.
Supplementary Table 68. CCS of B, Y, C and Z ions - ΔUA-GlcNS-I2S-GlcNS6S-UA-GlcNAc6S
Supplementary Table 69 CCS of B, Y, C and Z -SO_3 ions - ΔUA-GlcNS-I2S-GlcNS6S-UA-GlcNAc6S

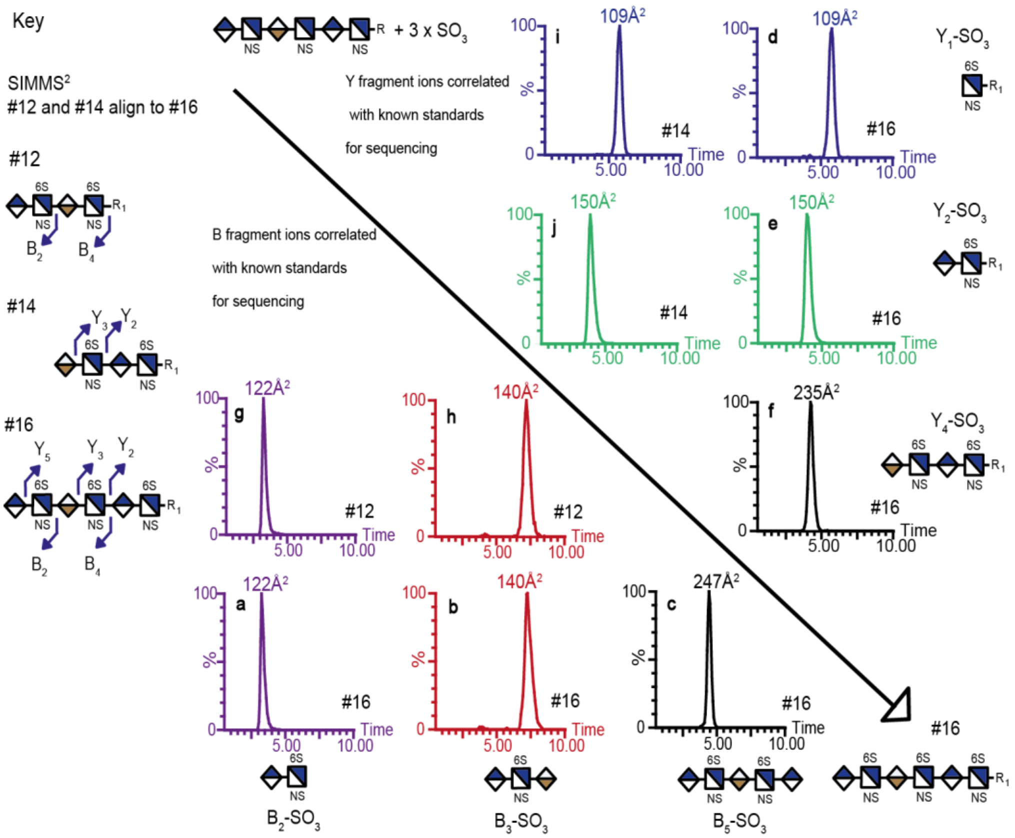

Supplementary Fig. 1. Using the SIMMS^2 method for sequencing a hexasaccharide. Overlaying two tetrasaccharide standard structures (#12 and #14) provides complete sequence coverage of the hexasaccharide (#16). Each structure was fragmented in the trap of the mass spectrometer and analysed using DTIMS, resulting in accurate CCS values. A comparison of overlapping fragment ions displayed the same CCS value between the two tetra- and hexasaccharides, providing a three-dimensional (MS, MS/MS and IMMS) sequencing method. **a-f**, The hexasaccharide #16 displays the DTIMS data to be determined. **g-h**, Tetrasaccharide #12 displayed CCS values from B -SO_3 ions; B_2-SO_3 – 122Å^2 and B_3-SO_3 – 140Å^2 matched CCS values observed in the hexasaccharide (a-c). **i-j**, Tetrasaccharide #14 displayed CCS values from Y ions; Y_1-SO_3 109Å^2 and Y_3-SO_3 150Å^2 matched the CCS values observed in the hexasaccharide **d-f**.

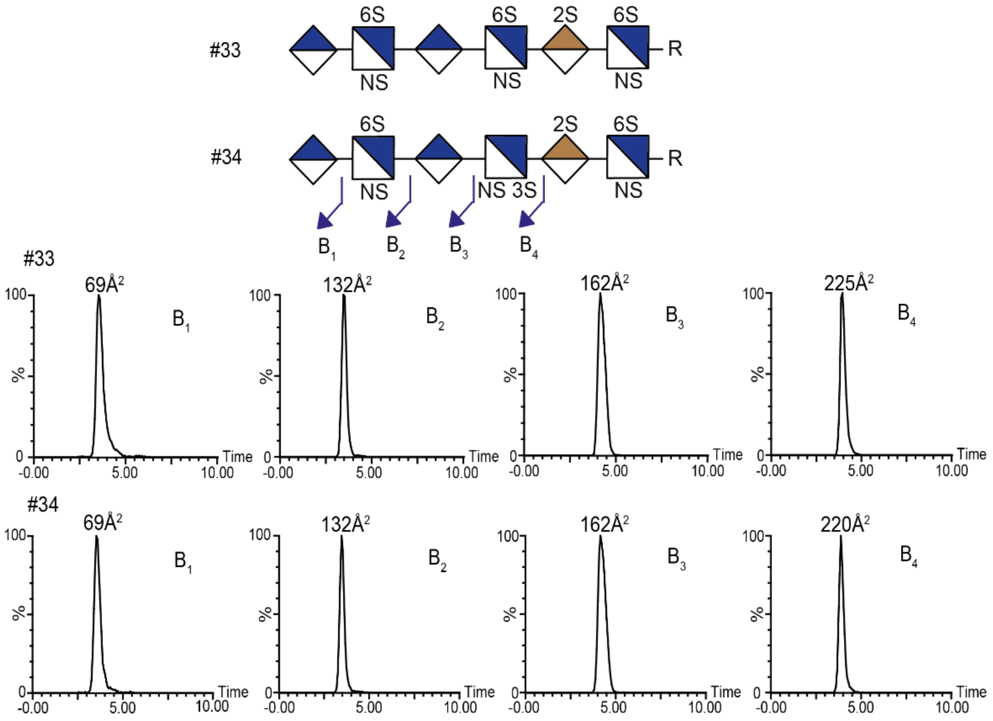

Supplementary Figure 2. SIMMS^2 sequencing of two hexasaccharides differing in a single 3*O*- and 6*O*-sulfation. HS structures #33 and #34 were fragmented in the trap and separated through IMMS to create a defined set of CCS values for each fragment. B ion fragments from #33 and #34 showed a difference at B_4 with the 6*O*-sulfated isomer (#33) showing a CCS value of 225 Å^2 and the B_4 fragment for 3*O*-sulfation (#34) demonstrating a CCS value of 220Å^2 respectively. Fragment ions from B/Y/C/Z are displayed in **Supplementary Tables 23 and 24.**

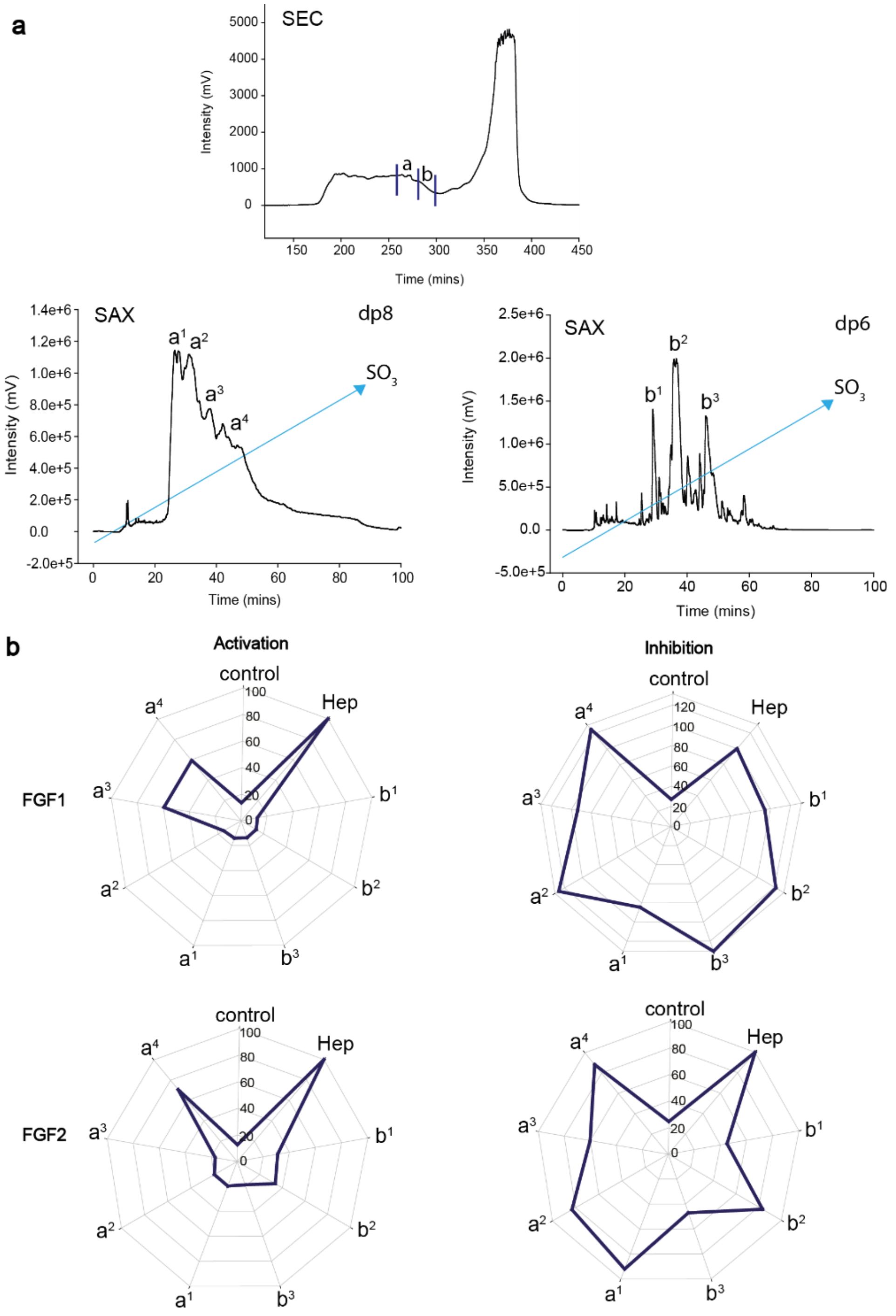

Supplementary Fig. 3. Purification of HS oligosaccharides with FGF1/2 bioactivity from porcine HS. a, Heparinase III digested porcine HS was separated by SEC and fractions a and b (corresponding to a dp8 and dp6, respectively) were further separated by SAX-HPLC to yield fractions $a^{1\text{-}4}$ and $b^{1\text{-}3}$. **b,** Radar charts illustrating BaF3 cell activation and inhibition of fractions $a^{1\text{-}4}$ and $b^{1\text{-}3}$. Activation assays were performed with FGF1 or FGF2 (1 ng/mL) and fractions as indicated. Heparin (3 μg/mL) was used as positive control, while FGF1 or FGF2 alone was used as negative. Inhibition assays were performed with the same fractions (3 μg/mL) in the presence of a sub-maximal dose of heparin (0.1 μg/mL). Cell proliferation results were expressed as a percentage of heparin activity set as 100%.

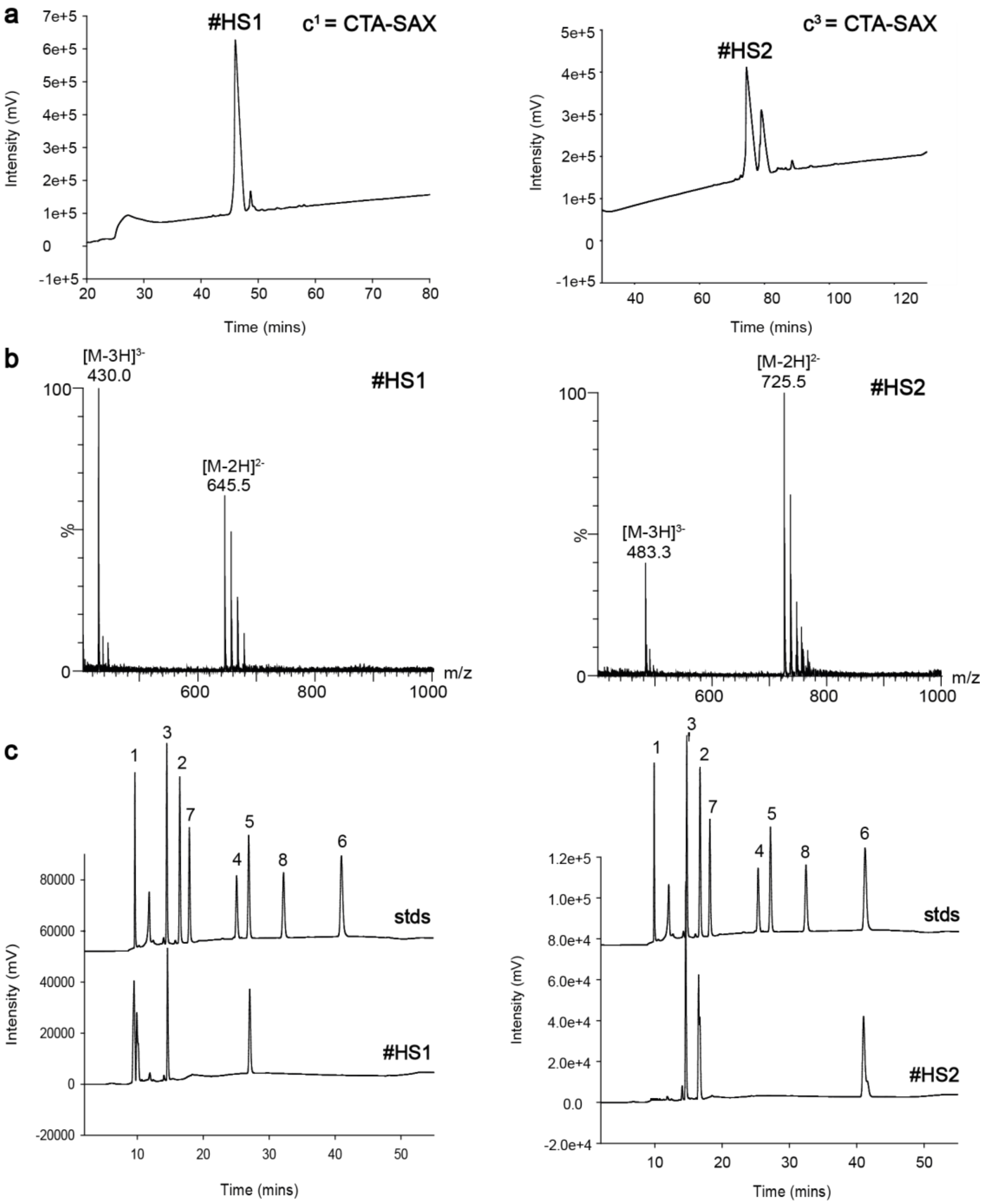

Supplementary Fig. 4. Isolation of pure hexasaccharides and their resulting disaccharide composition. a, Bioactive inhibitory hexasaccharides were purified further with CTA-SAX HPLC. **b,** Isolated CTA-SAX fractions #HS1 and #HS2 were subjected to mass spectrometry to confirm purity and mass. **c,** Disaccharide analysis of #HS1 and #HS2 through complete digestion to disaccharide products and separation on SAX ProPac PA1 (compared to authentic standards). Structure #HS1 contained ΔUA-GlcNAc, ΔUA-GlcNS and ΔUA2S-GlcNS, whereas structure #HS2 was composed of ΔUA-GlcNS, ΔUA-GlcNAc6S and ΔUA2S-GlcNS6S. Standards are 1- ΔUA-GlcNAc, 2 - ΔUA-GlcNAc6S, 3 - ΔUA-GlcNS, 4 - ΔUA-GlcNS6S, 5 – ΔUA2S-GlcNS, 6 – ΔUA2S-GlcNS6S, 7 – ΔUA2S-GlcNAc, 8 – ΔUA2S-GlcNAc6S.

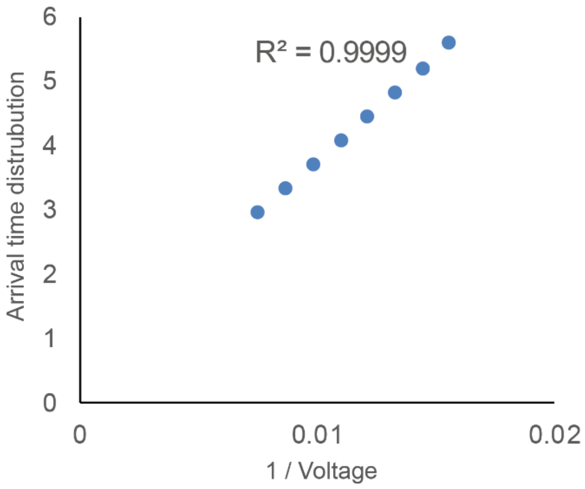

	Capillary - 0.6kV	Capillary - 0.8kV
Sample Cone - 2	210.33 (0.4)	210.60 (0.3)
Sample Cone - 20	210.70 (0.3)	210.38 (0.3)

Supplementary Fig. 5. CCS of #10 using different DTIMS source conditions. Standard #10 was sprayed at a capillary voltage of 0.6 kV and 0.8 kV and a sample cone voltage or 2 and 20. The CCS value of #10 was determined from 8 DTIMS voltage measurements.

Supplementary Table 1. Summary of the used library of standards.

No.	Chemdraw Structure	Structure[1]
**#1**	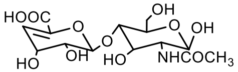	ΔUA-GlcNAc
**#2**	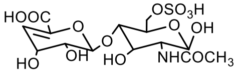	ΔUA-GlcNAc6S
**#3**	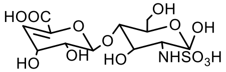	ΔUA-GlcNS
**#4**	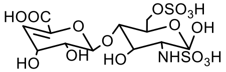	ΔUA-GlcNS6S
**#5**	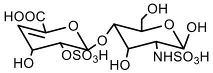	ΔUA2S-GlcNS
**#6**	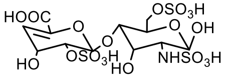	ΔUA2S-GlcNS6S
**#7**	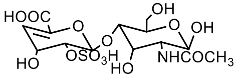	ΔUA2S-GlcNAc
**#8**	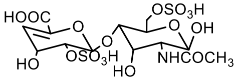	ΔUA2S-GlcNAc6S
**#9**	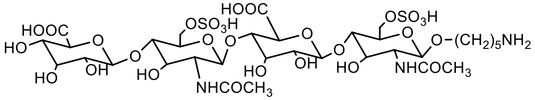	G-GlcNAc6S-G-GlcNAc6S-R_1

**#10**	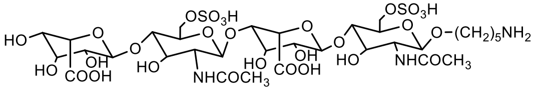	I-GlcNAc6S-I-GlcNAc6S-R$_1$
**#11**	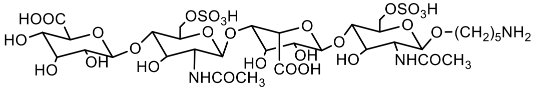	G-GlcNAc6S-I-GlcNAc6S-R$_1$
**#12**	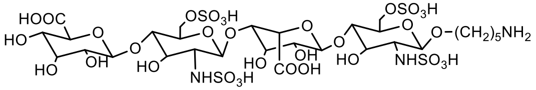	G-GlcNS6S-I-GlcNS6S-R$_1$
**#13**	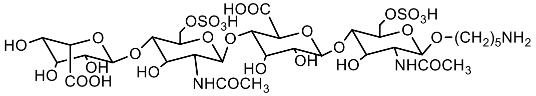	I-GlcNAc6S-G-GlcNAc6S-R$_1$
**#14**	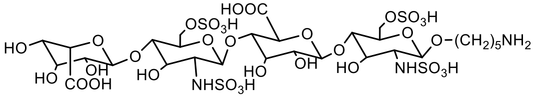	I-GlcNS6S-G-GlcNS6S-R$_1$
**#15**	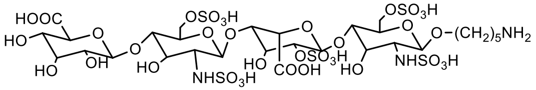	G-GlcNS6S-I2S-GlcNS6S-R$_1$
**#16**	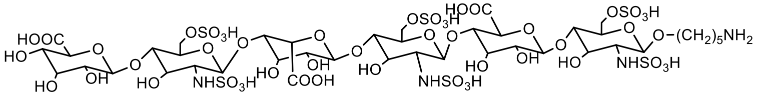	G-GlcNS6S-I-GlcNS6S-G-GlcNS6S-R$_1$
**#17**	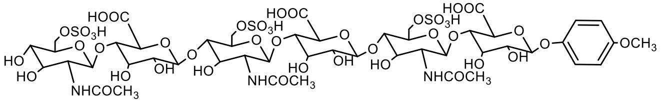	GlcNAc6S-[G-GlcNAc6S]$_2$-G-R$_2$
**#18**	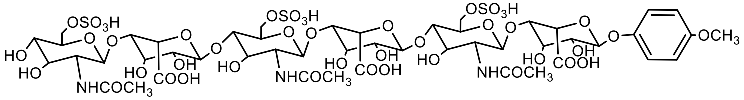	GlcNAc6S-[I-GlcNAc6S]$_2$-I-R$_2$
**#19**	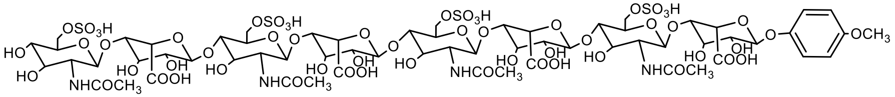	GlcNAc6S-[I-GlcNAc6S]$_3$-I-R$_2$
**#20**	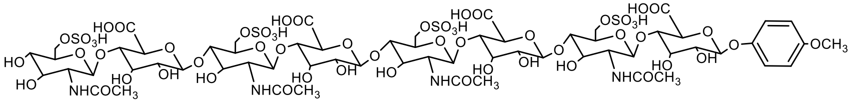	GlcNAc6S-[G-GlcNAc6S]$_3$-G-R$_2$

**#21**	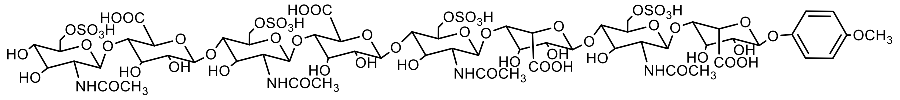	GlcNAc6S-G-GlcNAc6S-G-GlcNAc6S-I-GlcNAc6S-I-R_2
**#22**	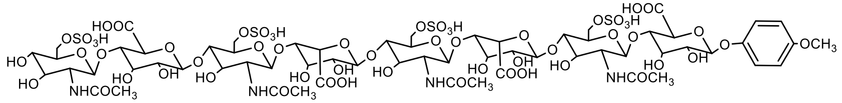	GlcNAc6S-G-GlcNAc6S-I-GlcNAc6S-I-GlcNAc6S-G-R_2
**#23**	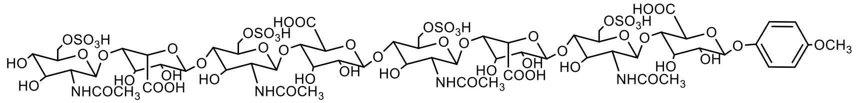	GlcNAc6S-I-GlcNAc6S-G-GlcNAc6S-I-GlcNAc6S-G-R_2
**#24**	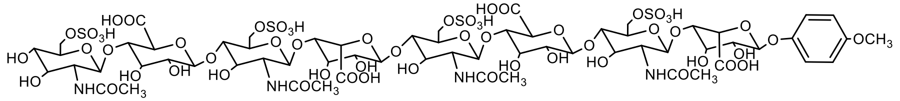	GlcNAc6S-G-GlcNAc6S-I-GlcNAc6S-G-GlcNAc6S-I-R_2
**#25**	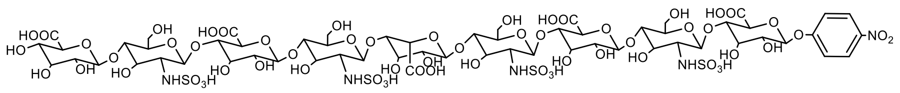	G-GlcNS-G-GlcNS-I-GlcNS-G-GlcNS-G-R_3
**#26**	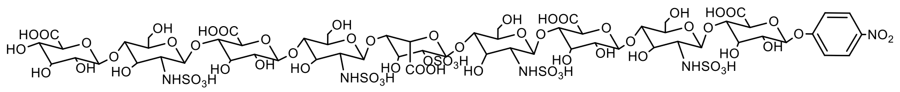	G-GlcNS-G-GlcNS-I2S-GlcNS-G-GlcNS-G-R_3
**#27**	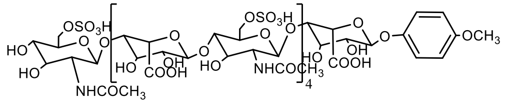	GlcNAc6S-[I-GlcNAc6S]$_4$-I-R_2
**#28**	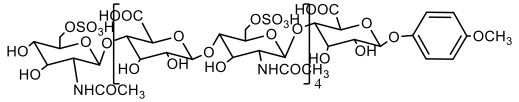	GlcNAc6S-[G-GlcNAc6S]$_4$-G-R_2
**#29**	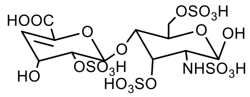	ΔUA2S-GlcNS3S6S
**#30**	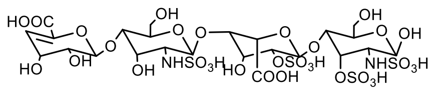	ΔUA-GlcNS-IdoA2S-GlcNS3S
**#31**	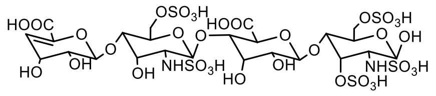	ΔUA-GlcNS6S-GlcA-GlcNS3S6S

**#32**	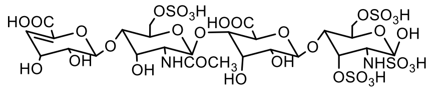	ΔUA-GlcNAc6S-GlcA-GlcNS3S6S
**#33**	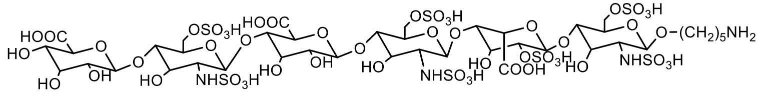	G-GlcNS6S-G-GlcNS6S-I2S-GlcNS6S-R_1
**#34**	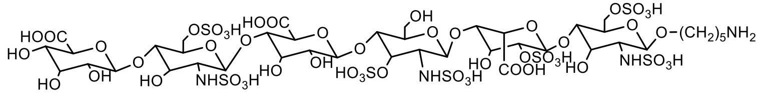	G-GlcNS6S-G-GlcNS3S-I2S-GlcNS6S-R_1
**#35**	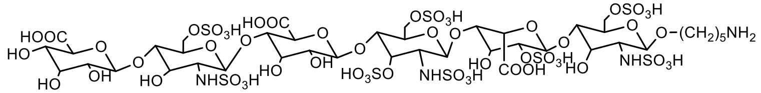	G-GlcNS6S-G-GlcNS3S6S-I2S-GlcNS6S-R_1
**#36**	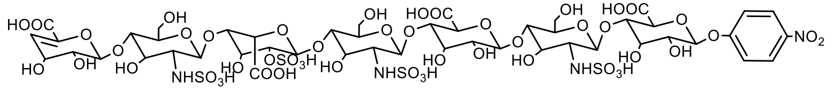	ΔUA-GlcNS-I2S-GlcNS-G-GlcNS-G-R_3

[1] The GlcA residues are represented by G and the IdoA residues represented by I. Tags R_1 is $(CH_2)_5NH_2$, R_2 is C_7H_7O and R_3 is $C_6H_4NO_2$.

[2] Standard #26 was digested with heparinase II and then separated using SAX, resulting in the purification of the dp7 + 2OS (#36) required.

Supplementary Table 2. Summary of CCS values obtained from the standard library.

	CCS (1-)[1]	CCS (2-)	CCS (3-)	CCS (4-)	CCS (5-)
**#1**	111.9 (0.3)[2]				
**#2**	121.0 (0.5)				
**#3**	112.4 (0.4)				
**#4**	123.5 (0.1)				
**#5**	124.3 (0.2)				
**#6**	137.4 (0.5)				
**#7**	123.6 (0.5)				
**#8**	134.2 (0.5)				
**#9**		206.2 (0.8)	214.2 (0.7)/247 (0.9)		
**#10**		210.1 (0.5)	242.0 (0.2)		
**#11**		206.8 (0.5)	232.4 (0.7)		
**#12**		200.1 (0.9)	236.2 (0.2)		
**#13**		208.7 (0.4)	245.5 (0.7)		
**#14**		201.5 (0.8)	234.0 (0.1)		
**#15**		203.9 (0.5)	238.8 (0.5)		
**#16**		255.6 (0.9)	258.8 (0.8)		
**#17**		266.8 (0.6)	296.5 (0.4)	347.4 (0.2)	
**#18**		265.8 (0.2)	292.4 (0.4)	342.2 (0.5)	
**#19**				379.8 (0.7)/416.0 (0.2)	433.8 (0.5)
**#20**				391.4 (0.8)	442.9 (0.2)
**#21**				379.2 (0.8)	438.5 (0.5)
**#22**				396.5 (0.7)	440.8 (0.5)
**#23**				400.8 (0.3)	435.6 (0.4)
**#24**				378.3 (0.5)	439.7 (0.4)
**#25**				437.4 (0.3)	438.4 (0.8)
**#26**				441.4 (0.3)	445.7 (0.5)
**#27**					499.8 (0.4)
**#28**					496.1 (0.7)
**#29**		149.7 (0.2)			
**#30**			228.0 (0.1)		
**#31**			228.9 (0.3)		
**#32**			234.5 (0.1)		
**#33**			280.5 (0.1)	339.5 (0.3)	
**#34**			282.4 (0.4)		
**#35**				328.3 (0.6)	
**#36**				363.2 (0.8)	

[1] Columns represent different charge states.

[2] Each CCS is an average of independent measurements with the corresponding standard deviation

Supplementary Table 3. CCS of B, Y, C and Z ions identified in isomeric tetrasaccharide UA-GlcNAc6S-UA-GlcNAc6S-R_1 structures, where R_1 is $(CH_2)_5NH_2$.

			**#9**		**#10**		**#11**		**#13**	
			G-GlcNAc6S-G-GlcNAc6S-R_1		I-GlcNAc6S-I-GlcNAc6S-R_1		G-GlcNAc6S-I-GlcNAc6S-R_1		I-GlcNAc6S-G-GlcNAc6S-R_1	
$[M-2H]^{2-}$			509.61	206.2 (0.8)[2]	509.61	210.1 (0.5)	509.61	206.8 (0.5)	509.61	208.7 (0.4)
$[M-3H]^{3-}$			339.4	214.2 (0.7)/247 (0.9)	339.4	242.0 (0.2)	339.4	232.4 (0.7)	339.4	245.5 (0.7)
	(1-)[1]	(2-)	(1-)[1]	(2-)	(1-)	(2-)	(1-)	(2-)	(1-)	(2-)
B1	175.02	87.01	x	x	x	x	x	x	x	x
B2	458.06	228.53	120.9 (0.3)	x	121.5 (0.5)	x	121.5 (0.3)	x	120.8 (0.1)	x
B3	634.09	316.54	150.6 (0.1)	x	150.7 (0.5)	x	150.7 (0.5)	x	150.5 (0.11)	x
B4	917.13	458.06	x	x	x	x	x	x	x	x
Y0	102.09	50.54	x	x	x	x	x	x	x	x
Y1	385.13	192.06	120.1 (0.5)	x	119.7 (0.6)	x	119.8 (0.2)	x	120.2 (0.5)	x
Y2	561.16	280.08	x	x	145.27 (0.1)	161.5 (0.3)	145.9 (0.4)	161.9 (0.2)	x	x
Y3	844.20	421.59	x	188.9 (0.2)	x	187.5 (0.5)	x	186.2 (0.7)	x	188.6 (0.3)
C1	193.03	96.01	x	x	x	x	x	x	x	x
C2	476.07	237.53	122.5 (0.2)	x	122.3 (0.2)	x	122.6 (0.3)	x	122.2 (0.2)	x
C3	652.10	325.55	x	x	x	x	x	x	x	x
C4	935.14	467.07	x	x	x	x	x	x	x	x
Z0	84.08	41.54	x	x	x	x	x	x	x	x
Z1	367.12	183.05	116.7 (0.2)	x	117.4 (0.3)	x	117.3 (0.2)	x	116.9 (0.3)	x
Z2	543.15	271.07	x	x	x	x	x	x	x	x
Z3	826.19	412.59	x	x	x	x	x	x	x	x

[1] Columns represent different charge states.

[2] Each CCS is an average of independent measurements with the corresponding standard deviation

Supplementary Table 4. CCS of B, Y, C and Z ions identified in isomeric tetra-saccharide UA-GlcNS6S-UA-GlcNS6S-R_1 structures, where R_1 is $(CH_2)_5NH_2$.

				**#12**			**#14**		
				G-GlcNS6S-I-GlcNS6S-R_1			I-GlcNS6S-G-GlcNS6S-R_1		
$[M-2H]^{2-}$				547.55	200.1 (0.9)[2]		547.55	201.5 (0.8)	
$[M-3H]^{3-}$				364.7	236.2 (0.2)		364.7	234.0 (0.1)	
	(1-)	(2-)	(3-)	(1-)[1]	(2-)	(3-)	(1-)	(2-)	(3-)
B1	175.02	87.01	57.67	68.9 (0.2)	x	x	69.0 (0.4)	x	x
B2	496.01	247.50	164.66	x	134.5 (0.6)	x	x	133.8 (0.3)	x
B3	672.04	335.52	223.34	x	160.2 (0.2)	x	x	165.5 (0.2)	x
B4	993.02	496.01	330.34	x	x	225.7 (0.2)	x	x	226.1 (0.2)
Y0	102.09	50.54	33.46	x	x	x	x	x	x
Y1	423.07	211.03	140.35	112.5 (0.3)	x	x	112.1 (0.1)	x	x
Y2	599.11	299.05	199.03	x	162.2 (0.5)	x	x	156.5 (0.5)	x
Y3	920.09	459.54	306.02	x	x	x	x	x	212.6 (0.6)
C1	193.03	96.01	63.67	x	x	x	x	x	x
C2	514.02	256.50	170.67	x	x	x	x	x	x
C3	690.05	344.52	229.34	x	x	x	x	165.6 (0.1)	x
C4	1011.03	505.01	336.34	x	x	x	x	x	x
Z0	84.08	41.54	27.36	x	x	x	x	x	x
Z1	405.06	202.03	134.35	x	x	x	x	x	x
Z2	581.10	290.04	193.03	x	x	x	x	x	x
Z3	902.08	450.54	300.02	x	x	x	x	x	x

[1] Columns represent different charge states.

[2] Each CCS is an average of independent measurements with the corresponding standard deviation

Supplementary Table 5. CCS of B, Y, C and Z ions identified in a tetra-saccharide structure (R_1 is $(CH_2)_5NH_2$).

**#15**						
G-GlcNS6S-I2S-GlcNS6S-R_1						
$[M-2H]^{2-}$	587.53	203.9 (0.5)[2]				
$[M-3H]^{3-}$	391.35	238.8 (0.5)				
	(1-)[1]	(2-)	(3-)	(1-)	(2-)	(3-)
B1	175.02	87.01	57.67	x	x	x
B2	496.01	247.50	164.66	x	135.2 (0.5)	x
B3	752.00	375.49	249.99	x	x	x
B4	1072.98	535.99	356.99	x	x	229.0 (0.4)
Y0	102.09	50.54	33.36	x	x	x
Y1	423.07	211.03	140.35	112.8 (0.3)	x	x
Y2	679.06	339.03	225.68	x	x	x
Y3	1000.05	499.52	332.68	x	x	x
C1	193.03	96.01	63.67	x	x	x
C2	514.02	256.50	170.67	x	x	x
C3	770.01	384.50	256.00	x	x	x
C4	1090.99	544.99	362.99	x	x	x
Z0	84.08	41.54	27.36	x	x	x
Z1	405.06	202.03	134.35	x	x	x
Z2	661.05	330.02	219.68	x	x	x
Z3	982.03	490.51	326.67	x	x	x

[1] Columns represent different charge states.

[2] Each CCS is an average of independent measurements with the corresponding standard deviation

Supplementary Table 6. CCS of B, Y, C and Z ions identified in a hexa-saccharide structure (R_1 is $(CH_2)_5NH_2$).

**#16**								
G-GlcNS6S-I-GlcNS6S-G-GlcNS6S-R_1								
$[M-2H]^{2-}$	796.06	255.6 (0.9)[2]						
$[M-3H]^{3-}$	530.37	258.8 (0.8)						
	(1-)[1]	(2-)	(3-)	(4-)	(1-)	(2-)	(3-)	(4-)
B1	175.02	87.01	57.67	43.00	x	x	x	x
B2	496.01	247.50	164.66	123.25	x	134.6 (0.4)	x	x
B3	672.04	335.52	223.34	167.25	x	160.3 (0.2)	x	x
B4	993.02	496.01	330.34	247.50	x	x	226.1 (0.2)	x
B5	1169.05	584.02	389.01	291.51	x	x	249.4 (0.8)	275.9 (0.7)
B6	1490.04	744.51	496.01	371.75	x	x	x	x
Y0	102.09	50.54	33.36	24.77	x	x	x	x
Y1	423.07	211.03	140.35	105.01	x	130.0 (0.7)	x	x
Y2	599.11	299.05	199.03	149.02	x	156.4 (0.5)	x	x
Y3	920.09	459.54	306.02	229.27	x	x	213.1 (0.7)	x
Y4	1096.12	547.56	364.70	273.27	x	x	233.2 (0.6)	x
Y5	1417.10	708.05	471.70	353.52	x	x	x	324.5 (0.2)
C1	193.03	96.01	63.67	47.50	x	x	x	x
C2	514.02	256.50	170.67	127.75	x	x	x	x
C3	690.05	344.52	229.34	171.76	x	x	x	x
C4	1011.03	505.01	336.34	252.00	x	x	x	x
C5	1187.06	593.03	395.02	296.01	x	x	x	x
C6	1508.05	753.52	502.01	376.26	x	x	x	x
Z0	84.08	41.54	27.36	20.26	x	x	x	x
Z1	405.06	202.03	134.35	100.51	x	x	x	x
Z2	581.10	290.04	193.03	144.52	x	x	x	x
Z3	902.08	450.54	300.02	224.76	x	x	x	x

Z4	1078.11	538.55	358.70	268.77	x	x	x	x
Z5	1399.09	699.04	465.69	349.02	x	x	x	x

[1] Columns represent different charge states.

[2] Each CCS is an average of independent measurements with the corresponding standard deviation

Supplementary Table 7. CCS of B, Y, C and Z ions identified in a hexa-saccharide GlcNAc6S-[G-GlcNAc6S]$_2$-G-R$_2$ structure, where R^2 is C_7H_7O.

**#17**						
GlcNAc6S-[G-GlcNAc6S]$_2$-G-R$_2$						
$[M-3H]^{3-}$	499.41	296.5 (0.4)[2]				
$[M-4H]^{4-}$	374.3	347.4 (0.2)				
	(1-)[1]	(2-)	(3-)	(1-)	(2-)	(3-)
B1	282.03	140.51	93.34	93.0 (0.2)	x	x
B2	458.06	228.53	152.01	120.8 (0.2)	135.1 (0.5)	x
B3	741.1	370.04	246.36	x	169.7 (0.6)	x
B4	917.13	458.06	305.04	x	x	225.6 (0.8)
B5	1200.16	599.58	399.38	x	x	270.4 (0.2)
B6	1376.2	687.59	458.06	x	x	291.3 (0.7)
Y0	122.04	60.51	40.01	x	x	x
Y1	299.08	149.03	99.02	105.4 (0.7)	x	x
Y2	582.11	290.55	193.37	154.4 (0.4)	166.0 (0.4)	x
Y3	758.14	378.57	252.04	x	x	x
Y4	1041.18	520.09	346.39	x	x	255.8 (0.5)
Y5	1217.21	608.1	405.07	x	x	x
C1	300.04	149.52	99.34	94.4 (0.2)	x	x
C2	476.07	237.53	158.02	122.3 (0.4)	x	x
C3	759.11	379.05	252.36	x	x	200.1 (0.9)
C4	935.14	467.07	311.04	x	185.2 (0.6)	x
C5	1218.18	608.58	405.39	x	x	273.9 (0.1)
C6	1394.21	696.6	464.06	x	x	x
Z0	105.03	52.01	34.34	x	x	x
Z1	281.07	140.03	93.02	x	x	x
Z2	564.1	281.55	187.36	x	x	x
Z3	740.13	369.56	246.04	x	x	x
Z4	1023.17	511.08	340.38	x	x	x
Z5	1199.2	599.1	399.06	x	x	x

[1] Columns represent different charge states.

[2] Each CCS is an average of independent measurements with the corresponding standard deviation

Supplementary Table 8. CCS of B, Y, C and Z ions identified in a hexa-saccharide GlcNAc6S-[I-GlcNAc6S]$_2$-I-R$_2$ structure, where R$_2$ is C_7H_7O.

**#18**						
GlcNAc6S-[I-GlcNAc6S]$_2$-I-R$_2$						
$[M-3H]^{3-}$	499.41	291.8 (0.7)[2]				
$[M-4H]^{4-}$	374.3	342.2 (0.3)				
	(1-)[1]	(2-)	(3-)	(1-)[1]	(2-)	(3-)
B1	282.03	140.51	93.34	92.9 (0.2)	x	x
B2	458.06	228.53	152.01	122.6 (0.4)	x	x
B3	741.10	370.04	246.36	x	176.0 (0.8)	x
B4	917.13	458.06	305.04	x	197.1 (0.17)	x
B5	1200.16	599.58	399.38	x	x	269.2 (0.8)
B6	1376.20	687.59	458.06	x	x	294.1 (0.16)
Y0	122.04	60.51	40.01	x	x	x
Y1	299.08	149.03	99.02	103.7 (0.1)	x	x
Y2	582.11	290.55	193.37	153.9 (0.6)	166.3 (0.3)	x
Y3	758.14	378.57	252.04	x	x	x
Y4	1041.18	520.09	346.39	x	x	x
Y5	1217.21	608.10	405.07	x	229.9 (0.1)	x
C1	300.04	149.52	99.34	94.1 (0.2)	x	x
C2	476.07	237.53	158.02	122.6 (0.3)	x	x
C3	759.11	379.05	252.36	x	x	x
C4	935.14	467.07	311.04	x	x	x
C5	1218.18	608.58	405.39	x	x	x
C6	1394.21	696.60	464.06	x	x	x
Z0	105.03	52.01	34.34	x	x	x
Z1	281.07	140.03	93.02	x	x	x
Z2	564.10	281.55	187.36	x	x	x
Z3	740.13	369.56	246.04	x	x	x
Z4	1023.17	511.08	340.38	x	x	x
Z5	1199.20	599.10	399.06	x	x	x

[1] Columns represent different charge states.

[2] Each CCS is an average of independent measurements with the corresponding standard deviation

Supplementary Table 9. CCS of B, Y, C and Z ions identified in an octa-saccharide GlcNAc6S-[I-GlcNAc6S]$_3$-I-R$_2$ structure, where R$_2$ is C_7H_7O.

**#19**								
GlcNAc6S-[I-GlcNAc6S]$_3$-I-R$_2$								
[M-4H]$^{4-}$	489.07	379.8 (0.7)[2]/416.0 (0.2)						
[M-5H]$^{5-}$	391.05	433.8 (0.5)						
[M-6H]$^{6-}$	325.7	449.4 (0.6)						
	(1-)[1]	(2-)	(3-)	(4-)	(1-)	(2-)	(3-)	(4-)
B1	282.03	140.51	93.34	69.75	93.3 (0.3)	x	x	x
B2	458.06	228.53	152.01	113.76	122.0 (0.6)	x	x	x
B3	741.10	370.04	246.36	184.52	x	176.3 (0.8)	x	x
B4	917.13	458.06	305.04	228.53	x	197.2 (0.7)	x	x
B5	1200.16	599.58	399.38	299.29	x	x	270.0 (0.3)	x
B6	1376.20	687.59	458.06	343.29	x	x	294.4 (0.7)	x
B7	1659.23	829.11	552.41	414.05	x	x	x	x
B8	1835.27	917.13	611.08	458.06	x	x	x	394.0 (0.6)
Y0	122.04	60.51	40.01	29.75	x	x	x	x
Y1	299.08	149.03	99.02	74.01	x	x	x	x
Y2	582.11	290.55	193.37	144.77	x	166.0 (0.6)	x	x
Y3	758.14	378.57	252.04	188.78	x	x	x	x
Y4	1041.18	520.09	346.39	259.54	x	x	x	x
Y5	1217.21	608.10	405.07	303.55	x	229.2 (0.2)	x	x
Y6	1500.25	749.62	499.41	374.31	x	290.5 (0.5)	x	x
Y7	1676.28	837.64	558.09	418.31	x	x	x	x
C1	300.04	149.52	99.34	74.25	94.0 (0.7)	x	x	x
C2	476.07	237.53	158.02	118.26	122.8 (0.5)	x	x	x
C3	759.11	379.05	252.36	189.02	x	x	x	x
C4	935.14	467.07	311.04	233.03	x	x	x	x

C5	1218.18	608.58	405.39	303.79	x	x	x	x
C6	1394.21	696.60	464.06	347.80	x	x	x	x
C7	1677.24	838.12	558.41	418.56	x	x	x	x
C8	1853.28	926.13	617.09	462.56	x	x	x	x
Z0	105.03	52.01	34.34	25.50	x	x	x	x
Z1	281.07	140.03	93.02	69.51	x	x	x	x
Z2	564.10	281.55	187.36	140.27	x	x	x	x
Z3	740.13	369.56	246.04	184.28	x	x	x	x
Z4	1023.17	511.08	340.38	255.04	x	x	x	x
Z5	1199.20	599.10	399.06	299.04	x	x	x	x
Z6	1482.24	740.62	493.41	369.80	x	x	x	x
Z7	1658.27	828.63	552.09	413.81	x	x	x	x

[1] Columns represent different charge states.

[2] Each CCS is an average of independent measurements with the corresponding standard deviation

Supplementary Table 10. CCS of B, Y, C and Z ions identified in an octa-saccharide GlcNAc6S-[G-GlcNAc6S]$_3$-G-R$_2$ structure, where R$_2$ is C_7H_7O.

**#20**								
GlcNAc6S-[G-GlcNAc6S]$_3$-G-R$_2$								
$[M-4H]^{4-}$	489.07	391.4 (0.8)[2]						
$[M-5H]^{5-}$	391.05	442.9 (0.2)						
$[M-6H]^{6-}$	325.7	456.7 (0.8)						
	(1-)[1]	(2-)	(3-)	(4-)	(1-)[1]	(2-)	(3-)	(4-)
B1	282.03	140.51	93.34	69.75	93.4 (0.1)	x	x	x
B2	458.06	228.53	152.01	113.76	121.6 (0.5)	x	x	x
B3	741.10	370.04	246.36	184.52	x	169.9 (0.4)		x
B4	917.13	458.06	305.04	228.53	x	194.1 (0.3)	225.8 (0.8)	x
B5	1200.16	599.58	399.38	299.29	x	x	270.5 (0.2)	x
B6	1376.20	687.59	458.06	343.29	x	x	x	x
B7	1659.23	829.11	552.41	414.05	x	x	291.0 (0.1)	322.1 (0.2)
B8	1835.27	917.13	611.08	458.06	x	x	x	391.8 (0.1)
Y0	122.04	60.51	40.01	29.75	x	x	x	x
Y1	299.08	149.03	99.02	74.01	x	x	x	x
Y2	582.11	290.55	193.37	144.77	154.3 (0.5)	165.8 (0.7)	x	x
Y3	758.14	378.57	252.04	188.78	x	x	x	x
Y4	1041.18	520.09	346.39	259.54	x	x	255.1 (0.8)	x
Y5	1217.21	608.10	405.07	303.55	x	x	x	x
Y6	1500.25	749.62	499.41	374.31	x	x	x	x
Y7	1676.28	837.64	558.09	418.31	x	x	x	x
C1	300.04	149.52	99.34	74.25	94.6 (0.4)	x	x	x
C2	476.07	237.53	158.02	118.26	122.2 (0.4)	x	x	x
C3	759.11	379.05	252.36	189.02	x	167.5 (0.1)	x	x
C4	935.14	467.07	311.04	233.03	x	x	x	x
C5	1218.18	608.58	405.39	303.79	x	x	x	x

C6	1394.21	696.60	464.06	347.80	x	x	309.1 (0.87)	x
C7	1677.24	838.12	558.41	418.56	x	x	x	x
C8	1853.28	926.13	617.09	462.56	x	x	x	x
Z0	105.03	52.01	34.34	25.50	x	x	x	x
Z1	281.07	140.03	93.02	69.51	x	x	x	x
Z2	564.10	281.55	187.36	140.27	x	x	x	x
Z3	740.13	369.56	246.04	184.28	x	x	x	x
Z4	1023.17	511.08	340.38	255.04	x	x	x	x
Z5	1199.20	599.10	399.06	299.04	x	x	x	x
Z6	1482.24	740.62	493.41	369.80	x	x	x	x
Z7	1658.27	828.63	552.09	413.81	x	x	x	x

[1] Columns represent different charge states.

[2] Each CCS is an average of independent measurements with the corresponding standard deviation

Supplementary Table 11. CCS of B, Y, C and Z ions identified in an octa-saccharide GlcNAc6S-G-GlcNac6S-G-GlcNAc6S-I-GlcNAc6S-I-R_2 structure, where R_2 is C_7H_7O.

**#21**								
GlcNAc6S-G-GlcNac6S-G-GlcNAc6S-I-GlcNAc6S-I-R_2								
$[M-4H]^{4-}$	489.07	379.2 (0.8)[2]						
$[M-5H]^{5-}$	391.05	438.5 (0.5)						
$[M-6H]^{6-}$	325.7	452.4 (0.8)						
	(1-)[1]	(2-)	(3-)	(4-)	(1-)	(2-)	(3-)	(4-)
B1	282.03	140.51	93.34	69.75	92.6 (0.5)	x	x	x
B2	458.06	228.53	152.01	113.76	121.4 (0.2)	x	x	x
B3	741.10	370.04	246.36	184.52	x	x	x	x
B4	917.13	458.06	305.04	228.53	x	x	224.8 (0.4)	x
B5	1200.16	599.58	399.38	299.29	x	x	270.1 (0.4)	x
B6	1376.20	687.59	458.06	343.29	x	x	x	322.3 (0.2)
B7	1659.23	829.11	552.41	414.05	x	x	x	367.3 (0.4)
B8	1835.27	917.13	611.08	458.06	x	x	x	x
Y0	122.04	60.51	40.01	29.75	x	x	x	x
Y1	299.08	149.03	99.02	74.01	103.4 (0.5)	x	x	x
Y2	582.11	290.55	193.37	144.77	153.8 (0.5)	x	x	x
Y3	758.14	378.57	252.04	188.78	x	x	x	x
Y4	1041.18	520.09	346.39	259.54	x	x	255.4 (0.6)	x
Y5	1217.21	608.10	405.07	303.55	x	x	x	x
Y6	1500.25	749.62	499.41	374.31	x	x	284.7 (0.6)	346.4 (0.7)
Y7	1676.28	837.64	558.09	418.31	x	x	x	x
C1	300.04	149.52	99.34	74.25	94.4 (0.3)	x	x	x
C2	476.07	237.53	158.02	118.26	122.4 (0.8)	x	x	x
C3	759.11	379.05	252.36	189.02	x	x	x	x
C4	935.14	467.07	311.04	233.03	x	x	x	x

C5	1218.18	608.58	405.39	303.79	x	x	x	x
C6	1394.21	696.60	464.06	347.80	x	x	x	x
C7	1677.24	838.12	558.41	418.56	x	x	x	x
C8	1853.28	926.13	617.09	462.56	x	x	x	x
Z0	105.03	52.01	34.34	25.50	x	x	x	x
Z1	281.07	140.03	93.02	69.51	x	x	x	x
Z2	564.10	281.55	187.36	140.27	x	x	x	x
Z3	740.13	369.56	246.04	184.28	x	x	x	x
Z4	1023.17	511.08	340.38	255.04	x	x	x	x
Z5	1199.20	599.10	399.06	299.04	x	x	x	x
Z6	1482.24	740.62	493.41	369.80	x	x	x	x
Z7	1658.27	828.63	552.09	413.81	x	x	x	x

[1] Columns represent different charge states.

[2] Each CCS is an average of independent measurements with the corresponding standard deviation

Supplementary Table 12. CCS of B, Y, C and Z ions identified in an octa-saccharide GlcNAc6S-G- GlcNac6S-I-GlcNAc6S-I-GlcNAc6S-G-R_2 structure, where R_2 is C_7H_7O.

**#22**								
GlcNAc6S-G-GlcNac6S-I-GlcNAc6S-I-GlcNAc6S-G-R_2								
$[M-4H]^{4-}$	489.07	396.5 (0.7)[2]						
$[M-5H]^{5-}$	391.05	440.8 (0.5)						
$[M-6H]^{6-}$	325.7	461.3 (0.7)						
	(1-)[1]	(2-)	(3-)	(4-)	(1-)	(2-)	(3-)	(4-)
B1	282.03	140.51	93.34	69.75	93.0 (0.2)	x	x	x
B2	458.06	228.53	152.01	113.76	121.6 (0.3)	x	x	x
B3	741.10	370.04	246.36	184.52	x	x	x	x
B4	917.13	458.06	305.04	228.53	x	x	x	x
B5	1200.16	599.58	399.38	299.29	x	x	272.8 (0.6)	x
B6	1376.20	687.59	458.06	343.29	x	x	x	323.2 (0.2)
B7	1659.23	829.11	552.41	414.05	x	x	x	x
B8	1835.27	917.13	611.08	458.06	x	x	x	x
Y0	122.04	60.51	40.01	29.75	x	x	x	x
Y1	299.08	149.03	99.02	74.01	x	x	x	x
Y2	582.11	290.55	193.37	144.77	154.9 (0.3)	x	x	x
Y3	758.14	378.57	252.04	188.78	x	x	x	x
Y4	1041.18	520.09	346.39	259.54	x	x	254.1 (0.28)	x
Y5	1217.21	608.10	405.07	303.55	x	x	x	300.6 (0.2)
Y6	1500.25	749.62	499.41	374.31	x	x	289.1 (0.6)	x
Y7	1676.28	837.64	558.09	418.31	x	x	x	x
C1	300.04	149.52	99.34	74.25	94.1 (0.3)	x	x	x
C2	476.07	237.53	158.02	118.26	123.0 (0.4)	x	x	x
C3	759.11	379.05	252.36	189.02	x	x	x	x
C4	935.14	467.07	311.04	233.03	x	x	x	x

C5	1218.18	608.58	405.39	303.79	x	x	x	x
C6	1394.21	696.60	464.06	347.80	x	x	x	x
C7	1677.24	838.12	558.41	418.56	x	x	x	371.4 (0.5)
C8	1853.28	926.13	617.09	462.56	x	x	x	x
Z0	105.03	52.01	34.34	25.50	x	x	x	x
Z1	281.07	140.03	93.02	69.51	x	x	x	x
Z2	564.10	281.55	187.36	140.27	x	x	x	x
Z3	740.13	369.56	246.04	184.28	x	x	x	x
Z4	1023.17	511.08	340.38	255.04	x	x	x	x
Z5	1199.20	599.10	399.06	299.04	x	x	x	x
Z6	1482.24	740.62	493.41	369.80	x	x	x	x
Z7	1658.27	828.63	552.09	413.81	x	x	x	x

[1] Columns represent different charge states.

[2] Each CCS is an average of independent measurements with the corresponding standard deviation

Supplementary Table 13. CCS of B, Y, C and Z ions identified in an octa-saccharide GlcNAc6S-I-GlcNAc6S-G-GlcNAc6S-I-GlcNAc6S-G-R_2 structure, where R_2 is C_7H_7O.

**#23**								
GlcNAc6S-I-GlcNAc6S-G-GlcNAc6S-I-GlcNAc6S-G-R_2								
$[M-4H]^{4-}$	489.07	400.8 (0.3)[2]						
$[M-5H]^{5-}$	391.05	435.6 (0.4)						
$[M-6H]^{6-}$	325.7	453.4 (0.7)						
	(1-)[1]	(2-)	(3-)	(4-)	(1-)	(2-)	(3-)	(4-)
B1	282.03	140.51	93.34	69.75	93.3 (0.6)	x	x	x
B2	458.06	228.53	152.01	113.76	x	x	x	x
B3	741.10	370.04	246.36	184.52	x	x	x	x
B4	917.13	458.06	305.04	228.53	x	x	x	x
B5	1200.16	599.58	399.38	299.29	x	x	272.0 (0.1)	x
B6	1376.20	687.59	458.06	343.29	x	x	x	x
B7	1659.23	829.11	552.41	414.05	x	x	x	x
B8	1835.27	917.13	611.08	458.06	x	x	x	x
Y0	122.04	60.51	40.01	29.75	x	x	x	x
Y1	299.08	149.03	99.02	74.01	x	x	x	x
Y2	582.11	290.55	193.37	144.77	155.0 (0.2)	x	x	x
Y3	758.14	378.57	252.04	188.78	x	x	x	x
Y4	1041.18	520.09	346.39	259.54	x	x	x	x
Y5	1217.21	608.10	405.07	303.55	x	x	x	x
Y6	1500.25	749.62	499.41	374.31	x	x	x	339.5 (0.3)
Y7	1676.28	837.64	558.09	418.31	x	x	x	366.4 (0.3)
C1	300.04	149.52	99.34	74.25	93.2 (0.1)	x	x	x
C2	476.07	237.53	158.02	118.26	122.0 (0.2)	132.8 (0.4)	x	x
C3	759.11	379.05	252.36	189.02	x	x	x	x
C4	935.14	467.07	311.04	233.03	x	x	x	x

C5	1218.18	608.58	405.39	303.79	x	x	x	x
C6	1394.21	696.60	464.06	347.80	x	x	x	315.3 (0.3)
C7	1677.24	838.12	558.41	418.56	x	x	x	367.2 (0.5)
C8	1853.28	926.13	617.09	462.56	x	x	x	x
Z0	105.03	52.01	34.34	25.50	x	x	x	x
Z1	281.07	140.03	93.02	69.51	x	x	x	x
Z2	564.10	281.55	187.36	140.27	x	x	x	x
Z3	740.13	369.56	246.04	184.28	x	x	x	x
Z4	1023.17	511.08	340.38	255.04	x	x	x	x
Z5	1199.20	599.10	399.06	299.04	x	x	x	x
Z6	1482.24	740.62	493.41	369.80	x	x	x	x
Z7	1658.27	828.63	552.09	413.81	x	x	x	x

[1] Columns represent different charge states.

[2] Each CCS is an average of independent measurements with the corresponding standard deviation

Supplementary Table 14. CCS of B, Y, C and Z ions identified in an octa-saccharide GlcNAc6S-G-GlcNAc6S-I-GlcNAc6S-G-GlcNAc6S-I-R_2 structure, where R_2 is C_7H_7O.

**#24**								
GlcNAc6S-G-GlcNac6S-I-GlcNAc6S-G-GlcNAc6S-I-R_2								
$[M-4H]^{4-}$	489.07	378.3 (0.5)[2]						
$[M-5H]^{5-}$	391.05	439.7 (0.4)						
$[M-6H]^{6-}$	325.7	451.4 (0.6)						
	(1-)[1]	(2-)	(3-)	(4-)	(1-)	(2-)	(3-)	(4-)
B1	282.03	140.51	93.34	69.75	93.1 (0.4)	x	x	x
B2	458.06	228.53	152.01	113.76	121.4 (0.6)	x	x	x
B3	741.10	370.04	246.36	184.52	x	170.5 (0.6)	x	x
B4	917.13	458.06	305.04	228.53	x	x	x	x
B5	1200.16	599.58	399.38	299.29	x	x	270.9 (0.8)	x
B6	1376.20	687.59	458.06	343.29	x	x	x	x
B7	1659.23	829.11	552.41	414.05	x	x	x	366.2 (0.5)
B8	1835.27	917.13	611.08	458.06	x	x	x	x
Y0	122.04	60.51	40.01	29.75	x	x	x	x
Y1	299.08	149.03	99.02	74.01	103.1 (0.4)	x	x	x
Y2	582.11	290.55	193.37	144.77	153.4 (0.3)	x	x	x
Y3	758.14	378.57	252.04	188.78	x	x	x	x
Y4	1041.18	520.09	346.39	259.54	x	x	x	x
Y5	1217.21	608.10	405.07	303.55	x	226.0 (0.5)	289.6 (0.6)	x
Y6	1500.25	749.62	499.41	374.31	x	288.4 (0.4)	x	x
Y7	1676.28	837.64	558.09	418.31	x	x	x	x
C1	300.04	149.52	99.34	74.25	94.1 (0.2)	x	x	x
C2	476.07	237.53	158.02	118.26	122.2 (0.2)	x	x	x
C3	759.11	379.05	252.36	189.02	x	x	x	x
C4	935.14	467.07	311.04	233.03	x	x	x	x

C5	1218.18	608.58	405.39	303.79	x	x	x	x
C6	1394.21	696.60	464.06	347.80	x	x	x	x
C7	1677.24	838.12	558.41	418.56	x	x	x	x
C8	1853.28	926.13	617.09	462.56	x	x	x	x
Z0	105.03	52.01	34.34	25.50	x	x	x	x
Z1	281.07	140.03	93.02	69.51	x	x	x	x
Z2	564.10	281.55	187.36	140.27	x	x	x	x
Z3	740.13	369.56	246.04	184.28	x	x	x	x
Z4	1023.17	511.08	340.38	255.04	x	x	x	x
Z5	1199.20	599.10	399.06	299.04	x	x	x	x
Z6	1482.24	740.62	493.41	369.80	x	x	x	x
Z7	1658.27	828.63	552.09	413.81	x	x	x	x

[1] Columns represent different charge states.

[2] Each CCS is an average of independent measurements with the corresponding standard deviation

Supplementary Table 15. CCS of B, Y, C and Z ions identified in a 9mer, G-GlcNS-G-GlcNS-I-GlcNS-G-GlcNS-G-R_3 structure, where R_3 is $C_6H_4NO_2$.

**#25**								
G-GlcNS-G-GlcNS-I-GlcNS-G-GlcNS-G-R_3								
$[M-4H]^{4-}$	494.81	437.4 (0.3)[2]						
$[M-5H]^{5-}$	395.65	438.4 (0.8)						
	(1-)[1]	(2-)	(3-)	(4-)	(1-)	(2-)	(3-)	(4-)
B1	175.02	87.01	57.67	43.00	71.5 (0.3)	x	x	x
B2	416.05	207.52	138.01	103.26	112.4 (0.4)	x	x	x
B3	592.08	295.54	196.69	147.26	x	157.8 (0.4)	x	x
B4	833.11	416.05	277.03	207.52	x	197.4 (0.4)	x	x
B5	1009.14	504.07	335.71	251.53	x	235.2 (0.5)	x	x
B6	1250.17	624.58	416.05	311.79	x	x	x	x
B7	1426.20	712.59	474.73	355.79	x	x	321.4 (0.4)	x
B8	1667.22	833.11	555.07	416.05	x	x	x	x
B9	1843.25	921.12	613.75	460.06	x	x	x	407.6 (0.5)
Y0	138.02	68.51	45.33	33.75	x	x	x	x
Y1	314.05	156.52	104.01	77.76	x	x	x	x
Y2	555.08	277.03	184.35	138.01	x	x	x	x
Y3	731.11	365.05	243.03	182.02	x	x	x	x
Y4	972.13	485.56	323.37	242.28	x	x	x	x
Y5	1148.17	573.58	382.05	286.29	x	x	x	x
Y6	1389.19	694.09	462.39	346.54	x	x	x	x
Y7	1565.22	782.11	521.07	390.55	x	x	x	x
Y8	1806.25	902.62	601.41	450.81	x	x	x	x
C1	192.03	95.51	63.34	47.25	x	x	x	x
C2	433.05	216.02	143.68	107.51	x	x	x	x
C3	609.08	304.04	202.36	151.52	x	x	x	x
C4	850.11	424.55	282.70	211.77	x	x	x	x

C5	1026.14	512.57	341.38	255.78	x	x	x	x
C6	1267.17	633.08	421.72	316.04	x	x	x	x
C7	1443.20	721.10	480.39	360.04	x	x	x	x
C8	1684.23	841.61	560.74	420.30	x	x	x	x
C9	1860.26	929.62	619.41	464.31	x	x	x	x
Z0	120.01	59.50	39.33	29.25	x	x	x	x
Z1	296.04	147.52	98.01	73.25	x	x	x	x
Z2	537.07	268.03	178.35	133.51	x	x	x	x
Z3	713.10	356.05	237.03	177.52	x	x	x	x
Z4	954.12	476.56	317.37	237.78	x	x	x	x
Z5	1130.16	564.57	376.05	281.78	x	x	x	x
Z6	1371.18	685.09	456.39	342.04	x	x	x	x
Z7	1547.21	773.10	515.07	386.05	x	x	x	x
Z8	1788.24	893.62	595.41	446.30	x	x	x	x

[1] Columns represent different charge states.

[2] Each CCS is an average of independent measurements with the corresponding standard deviation

Supplementary Table 16. CCS of B, Y, C and Z ions identified in a dp9 + 1x2*O*-sulfate, G-GlcNS-G-GlcNS-I2S-GlcNS-G-GlcNS-G-R_3 structure, where R_3 is $C_6H_4NO_2$.

**#26**								
G-GlcNS-G-GlcNS-I2S-GlcNS-G-GlcNS-G-R_3								
$[M-4H]^{4-}$	514.8	441.4 (0.3)[2]						
$[M-5H]^{5-}$	411.64	445.7 (0.5)						
	(1-)[1]	(2-)	(3-)	(4-)	(1-)	(2-)	(3-)	(4-)
B1	175.02	87.01	57.67	43.00	x	x	x	x
B2	416.05	207.52	138.01	103.26	112.2 (0.2)	x	x	x
B3	592.08	295.54	196.69	147.26	x	158.0 (0.2)	x	x
B4	833.11	416.05	277.03	207.52	x	197.3 (0.6)	x	x
B5	1089.10	544.04	362.36	271.52	x	x	249.6 (0.7)	x
B6	1330.12	664.56	442.70	331.77	x	x	x	x
B7	1506.15	752.57	501.38	375.78	x	x	330.0 (0.6)	x
B8	1747.18	873.09	581.72	436.04	x	x	x	379.0 (0.4)
B9	1923.21	961.10	640.40	480.05	x	x	x	405.5 (0.2)
Y0	138.02	68.51	45.33	33.75	x	x	x	x
Y1	314.05	156.52	104.01	77.76	x	x	x	x
Y2	555.08	277.03	184.35	138.01	x	x	x	x
Y3	731.11	365.05	243.03	182.02	x	x	x	x
Y4	972.13	485.56	323.37	242.28	x	x	x	x
Y5	1228.12	613.56	408.70	306.27	x	x	x	x
Y6	1469.15	734.07	489.04	366.53	x	x	x	x
Y7	1645.18	822.09	547.72	410.54	x	x	x	x
Y8	1886.21	942.60	628.06	470.80	x	x	x	x
C1	192.03	95.51	63.34	47.25	x	x	x	x
C2	433.05	216.02	143.68	107.51	x	x	x	x
C3	609.08	304.04	202.36	151.52	x	x	x	x

C4	850.11	424.55	282.70	211.77	x	x	x	x
C5	1106.10	552.55	368.03	275.77	x	x	x	x
C6	1347.12	673.06	448.37	336.03	x	x	x	x
C7	1523.16	761.07	507.05	380.03	x	x	x	x
C8	1764.18	881.59	587.39	440.29	x	x	x	x
C9	1940.21	969.60	646.07	484.30	x	x	x	x
Z0	120.01	59.50	39.33	29.25	x	x	x	x
Z1	296.04	147.52	98.01	73.25	x	x	x	x
Z2	537.07	268.03	178.35	133.51	x	x	x	x
Z3	713.10	356.05	237.03	177.52	x	x	x	x
Z4	954.12	476.56	317.37	237.78	x	x	x	x
Z5	1210.11	604.55	402.70	301.77	x	x	x	x
Z6	1451.14	725.07	483.04	362.03	x	x	x	x
Z7	1627.17	813.08	541.72	406.04	x	x	x	x
Z8	1868.20	933.59	622.06	466.29	x	x	x	x

[1] Columns represent different charge states.

[2] Each CCS is an average of independent measurements with the corresponding standard deviation

Supplementary Table 17. CCS of B, Y, C and Z ions identified in a dp10 GlcNAc6S-[I-GlcNAc6S]$_4$-I-R$_2$ structure, where R$_2$ is C_7H_7O.

#27								
GlcNAc6S-[I-GlcNAc6S]$_4$-I-R$_2$								
$[M-5H]^{5-}$	482.87	499.8 (0.4)[2]						
$[M-6H]^{6-}$	402.22	536.4 (0.7)						
	(1-)[1]	(2-)	(3-)	(4-)	(1-)	(2-)	(3-)	(4-)
B1	282.03	140.51	93.34	69.75	92.9 (0.5)	x	x	x
B2	458.06	228.53	152.01	113.76	121.8 (0.6)	x	x	x
B3	741.10	370.04	246.36	184.52	x	176.0 (0.7)	x	x
B4	917.13	458.06	305.04	228.53	x	197.2 (0.5)	x	x
B5	1200.16	599.58	399.38	299.29	x	x	269.1 (0.3)	x
B6	1376.20	687.59	458.06	343.29	x	x	294.6 (0.2)	x
B7	1659.23	829.11	552.41	414.05	x	x	x	x
B8	1835.27	917.13	611.08	458.06	x	x	x	394.0 (0.6)
B9	2118.30	1058.65	705.43	528.82	x	x	x	x
B10	2294.33	1146.66	764.11	572.83	x	x	x	x
Y0	123.04	61.02	40.34	30.01	69.3 (0.1)	x	x	x
Y1	299.08	149.03	99.02	74.01	x	x	x	x
Y2	582.11	290.55	193.37	144.77	x	166.3 (0.8)	x	x
Y3	758.14	378.57	252.04	188.78	x	x	x	x
Y4	1041.18	520.09	346.39	259.54	x	x	x	x
Y5	1217.21	608.10	405.07	303.55	x	x	x	x
Y6	1500.25	749.62	499.41	374.31	x	x	x	x
Y7	1676.28	837.64	558.09	418.31	x	x	368.5 (0.9)	x
Y8	1959.32	979.15	652.43	489.07	x	x	x	x
Y9	2135.35	1067.17	711.11	533.08	x	x	x	x
C1	300.04	149.52	99.34	74.25	x	x	x	x
C2	476.07	237.53	158.02	118.26	x	x	x	x

C3	759.11	379.05	252.36	189.02	x	x	x	x
C4	935.14	467.07	311.04	233.03	x	x	x	x
C5	1218.18	608.58	405.39	303.79	x	x	x	x
C6	1394.21	696.60	464.06	347.80	x	x	x	x
C7	1677.24	838.12	558.41	418.56	x	x	x	x
C8	1853.28	926.13	617.09	462.56	x	x	x	x
C9	2136.31	1067.65	711.43	533.32	x	x	x	x
C10	2312.34	1155.67	770.11	577.33	x	x	x	x
Z0	105.03	52.01	34.34	25.50	x	x	x	x
Z1	281.07	140.03	93.02	69.51	x	x	x	x
Z2	564.10	281.55	187.36	140.27	x	x	x	x
Z3	740.13	369.56	246.04	184.28	x	x	x	x
Z4	1023.17	511.08	340.38	255.04	x	x	x	x
Z5	1199.20	599.10	399.06	299.04	x	x	x	x
Z6	1482.24	740.62	493.41	369.80	x	x	x	x
Z7	1658.27	828.63	552.09	413.81	x	x	x	x
Z8	1941.31	970.15	646.43	484.57	x	x	x	x
Z9	2117.34	1058.17	705.11	528.58	x	x	x	x

[1] Columns represent different charge states.

[2] Each CCS is an average of independent measurements with the corresponding standard deviation

Supplementary Table 18. CCS of B, Y, C and Z ions identified in a dp10 GlcNAc6S-[G-GlcNAc6S]$_4$-G-R$_2$ structure, where R$_2$ is C_7H_7O.

**#28**								
GlcNAc6S-[G-GlcNAc6S]$_4$-G-R$_2$								
$[M-5H]^{5-}$	482.87	496.1 (0.7)[2]						
$[M-6H]^{6-}$	402.22	524.5 (0.2)						
	(1-)[1]	(2-)	(3-)	(4-)	(1-)	(2-)	(3-)	(4-)
B1	282.03	140.51	93.34	69.75	93.2 (0.2)	x	x	x
B2	458.06	228.53	152.01	113.76	121.3 (0.8)	x	x	x
B3	741.10	370.04	246.36	184.52	x	x	x	x
B4	917.13	458.06	305.04	228.53	x	194.2 (0.2)	225.5 (0.4)	x
B5	1200.16	599.58	399.38	299.29	x	x	x	x
B6	1376.20	687.59	458.06	343.29	x	x	291.6 (0.2)	x
B7	1659.23	829.11	552.41	414.05	x	x	x	x
B8	1835.27	917.13	611.08	458.06	x	x	x	390.9 (0.6)
B9	2118.30	1058.65	705.43	528.82	x	x	x	x
B10	2294.33	1146.66	764.11	572.83	x	x	x	x
Y0	123.04	61.02	40.34	30.01	69.0 (0.3)	x	x	x
Y1	299.08	149.03	99.02	74.01	x	x	x	x
Y2	582.11	290.55	193.37	144.77	x	x	x	x
Y3	758.14	378.57	252.04	188.78	x	x	x	x
Y4	1041.18	520.09	346.39	259.54	x	x	x	x
Y5	1217.21	608.10	405.07	303.55	x	x	281.0 (0.6)	x
Y6	1500.25	749.62	499.41	374.31	x	x	x	x
Y7	1676.28	837.64	558.09	418.31	x	x	x	x
Y8	1959.32	979.15	652.43	489.07	x	x	x	x
Y9	2135.35	1067.17	711.11	533.08	x	x	x	x
C1	300.04	149.52	99.34	74.25	x	x	x	x
C2	476.07	237.53	158.02	118.26	x	x	x	x

C3	759.11	379.05	252.36	189.02	x	x	x	x
C4	935.14	467.07	311.04	233.03	x	x	x	x
C5	1218.18	608.58	405.39	303.79	x	x	x	x
C6	1394.21	696.60	464.06	347.80	x	x	x	x
C7	1677.24	838.12	558.41	418.56	x	x	x	x
C8	1853.28	926.13	617.09	462.56	x	x	x	x
C9	2136.31	1067.65	711.43	533.32	x	x	x	x
C10	2312.34	1155.67	770.11	577.33	x	x	x	x
Z0	105.03	52.01	34.34	25.50	x	x	x	x
Z1	281.07	140.03	93.02	69.51	x	x	x	x
Z2	564.10	281.55	187.36	140.27	x	x	x	x
Z3	740.13	369.56	246.04	184.28	x	x	x	x
Z4	1023.17	511.08	340.38	255.04	x	x	x	x
Z5	1199.20	599.10	399.06	299.04	x	x	x	x
Z6	1482.24	740.62	493.41	369.80	x	x	x	x
Z7	1658.27	828.63	552.09	413.81	x	x	x	x
Z8	1941.31	970.15	646.43	484.57	x	x	x	x
Z9	2117.34	1058.17	705.11	528.58	x	x	x	x

[1] Columns represent different charge states.

[2] Each CCS is an average of independent measurements with the corresponding standard deviation

Supplementary Table 19. CCS of B, Y, C and Z ions identified in disaccharide ΔUA2S-GlcNS3S6S structure. Error +/- values are donated in brackets.

**#29**				
ΔUA2S-GlcNS3S6S				
$[M-2H]^{2-}$	327.45	149.7 (0.2)		
	(1-)[1]	(2-)	(1-)	(2-)
B1	236.97	117.98	79.7 (0.3)	x
Y0	417.94	208.47	x	x
C1	254.98	126.99	138.6 (0.2)/162.2 (0.7)	x
Z0	399.93	199.46	x	x

[1] Columns represent different charge states.

[2] Each CCS is an average of independent measurements with the corresponding standard deviation

Supplementary Table 20. CCS of B, Y, C and Z ions identified in tetrasaccharide ΔUA-GlcNS-IdoA2S-GlcNS3S structure. Error +/- values are donated in brackets.

**#30**						
ΔUA-GlcNS-IdoA2S-GlcNS3S						
$[M-3H]^{3-}$	330.33	228.0 (0.1)				
	(1-)[1]	(2-)	(3-)	(1-)	(2-)	(3-)
B1	157.01	78.00	51.67	70.7 (0.4)	x	x
B2	398.04	198.52	132.01	x	119.9 (0.5)	x
B3	654.03	326.51	217.34	x	162.4 (0.3)	x
B4	975.01	487.00	324.33	x	x	218.1 (0.7)
Y0	337.99	168.49	111.99	x	104.9 (0.1)	x
Y1	593.97	296.48	197.32	x	x	x
Y2	835.00	417.00	277.66	x	x	x
C1	175.02	87.01	57.67	71.5 (0.5)	x	x
C2	416.05	207.52	138.01	113.9 (0.1)	120.8 (0.5)	x
C3	672.04	335.52	223.34	147.16 (0.5)	x	x
Z0	319.97	159.48	105.99	x	x	x
Z1	575.96	287.48	191.32	x	x	x
Z2	816.99	407.99	271.66	x	x	x

[1] Columns represent different charge states.

[2] Each CCS is an average of independent measurements with the corresponding standard deviation

Supplementary Table 21. CCS of B, Y, C and Z ions identified in tetrasaccharide ΔUA-GlcNS6S-GlcA-GlcNS3S6S structure. Error +/- values are donated in brackets.

**#31**				
ΔUA-GlcNS6S-G-GlcNS3S6S				
$[M-3H]^{3-}$	356.98	228.9 (0.3)		
	(1-)[1]	(2-)	(1-)	(2-)
B1	157.01	78.00	70.0 (0.5)	x
B2	478.00	238.49	118.0 (0.1)	128.5 (0.1)
B3	654.03	326.51	x	163.4 (0.1)
Y0	417.94	208.47	x	x
Y1	593.97	296.48	x	x
Y2	914.96	456.97	x	x
C1	175.02	87.01	71.6 (0.5)	x
C2	496.01	247.50	x	131.8 (0.4)
C3	672.04	335.52	x	164.7 (0.2)
Z0	399.93	199.46	x	x
Z1	575.96	287.48	x	x
Z2	896.95	447.97	x	x

[1] Columns represent different charge states.

[2] Each CCS is an average of independent measurements with the corresponding standard deviation

Supplementary Table 22. CCS of B, Y, C and Z ions identified in tetrasaccharide ΔUA-GlcNAc6S-GlcA-GlcNS3S6S structure. Error +/- values are donated in brackets.

**#32**				
ΔUA-GlcNAc6S-GlcA-GlcNS3S6S				
$[M-3H]^{3-}$	344.33	234.5 (0.1)		
	(1-)[1]	(2-)	(1-)	(2-)
B1	157.01	78.00	69.7 (0.1)	x
B2	440.05	219.52	117.7 (0.1)	132.4 (0.8)
B3	616.08	307.54	151.7 (0.1)	x
Y0	417.94	208.47	x	x
Y1	593.97	296.48	x	x
Y2	877.01	438.00	x	x
C1	175.02	87.01	71.5 (0.5)	x
C2	458.06	228.53	120.6 (0.1)	131.5 (0.1)
C3	634.09	316.54	154.3 (0.2)	x
Z0	399.93	199.46	x	x
Z1	575.96	287.48	x	x
Z2	859.00	429.00	x	x

[1] Columns represent different charge states.

[2] Each CCS is an average of independent measurements with the corresponding standard deviation

Supplementary Table 23. CCS of B, Y, C and Z ions identified in hexasaccharide G-GlcNS6S-G-GlcNS6S-I2S-GlcNS6S-R_1 structure, where R_1 is $(CH_2)_5NH_2$.

**#33**						
G-GlcNS6S-G-GlcNS6S-I2S-GlcNS6S-R_1						
$[M-3H]^{3-}$	557.06	280.5 (0.1)				
$[M-4H]^{4-}$	417.52	339.5 (0.3)				
	(1-)[1]	(2-)	(3-)	(1-)	(2-)	(3-)
B1	175.02	87.01	57.67	69.0 (0.4)	x	x
B2	496.01	247.50	164.66	x	132.5 (0.5)	x
B3	672.04	335.52	223.34	x	161.7 (0.5)	x
B4	993.02	496.01	330.34	x	x	225.1 (0.7)
B5	1249.01	624.00	415.66	x	x	x
B6	1569.99	784.49	522.66	x	x	x
Y0	102.09	50.54	33.36	x	x	x
Y1	423.07	211.03	140.35	x	128.8 (0.7)	x
Y2	679.06	339.03	225.68	x	x	x
Y3	1000.05	499.52	332.68	x	x	x
Y4	1176.08	587.53	391.35	x	x	x
Y5	1497.06	748.03	498.35	x	x	x
C1	193.03	96.01	63.67	74.2 (0.4)	x	x
C2	514.02	256.50	170.67	x	133.7 (0.5)	x
C3	690.05	344.52	229.34	x	x	x
C4	1011.03	505.01	336.34	x	x	x
C5	1267.02	633.01	421.67	x	x	x
C6	1588.00	793.50	528.66	x	x	x
Z0	84.08	41.54	27.36	x	x	x
Z1	405.06	202.03	134.35	x	x	x
Z2	661.05	330.02	219.68	x	x	x
Z3	982.03	490.51	326.67	x	x	x
Z4	1158.07	578.53	385.35	x	x	x
Z5	1479.05	739.02	492.34	x	x	x

[1] Columns represent different charge states.

[2] Each CCS is an average of independent measurements with the corresponding standard deviation

Supplementary Table 24. CCS of B, Y, C and Z ions identified in hexasaccharide G-GlcNS6S-G-GlcNS3S-I2S-GlcNS6S-$(CH_2)_5NH_2$ structure, where R_1 is $(CH_2)_5NH_2$.

**#34**						
G-GlcNS6S-G-GlcNS3S-I2S-GlcNS6S-R_1						
$[M-3H]^{3-}$	557.06	282.4 (0.4)				
	(1-)[1]	(2-)	(3-)	(1-)	(2-)	(3-)
B1	175.02	87.01	57.67	68.8 (0.2)	x	x
B2	496.01	247.50	164.66	x	132.2 (0.5)	x
B3	672.04	335.52	223.34	x	162.1 (0.4)	x
B4	993.02	496.01	330.34	x	x	220.4 (0.5)
B5	1249.01	624.00	415.66	x	x	x
B6	1569.99	784.49	522.66	x	x	x
Y0	102.09	50.54	33.36	x	x	x
Y1	423.07	211.03	140.35	x	128.9 (0.5)	x
Y2	679.06	339.03	225.68	x	x	x
Y3	1000.05	499.52	332.68	x	x	x
Y4	1176.08	587.53	391.35	x	x	x
Y5	1497.06	748.03	498.35	x	x	x
C1	193.03	96.01	63.67	73.8 (0.4)	x	x
C2	514.02	256.50	170.67	x	133.9 (0.6)	x
C3	690.05	344.52	229.34	x	x	x
C4	1011.03	505.01	336.34	x	x	x
C5	1267.02	633.01	421.67	x	x	x
C6	1588.00	793.50	528.66	x	x	x
Z0	84.08	41.54	27.36	x	x	x
Z1	405.06	202.03	134.35	x	x	x
Z2	661.05	330.02	219.68	x	x	x
Z3	982.03	490.51	326.67	x	x	x
Z4	1158.07	578.53	385.35	x	x	x
Z5	1479.05	739.02	492.34	x	x	x

[1] Columns represent different charge states.

[2] Each CCS is an average of independent measurements with the corresponding standard deviation

Supplementary Table 25. CCS of B, Y, C and Z ions identified in hexasaccharide G-GlcNS6S-G-GlcNS6S3S-I2S-GlcNS6S-R_1, where R_1 is $(CH_2)_5NH_2$.

#35						
G-GlcNS6S-G-GlcNS6S3S-I2S-GlcNS6S-R_1						
$[M-4H]^{4-}$	437.51	328.3 (0.6)				
	(1-)[1]	(2-)	(3-)	(1-)	(2-)	(3-)
B1	175.02	87.01	57.67	69.0 (0.2)	x	x
B2	496.01	247.50	164.66	x	132.8 (0.4)	x
B3	672.04	335.52	223.34	x	162.4 (0.4)	x
B4	1072.98	535.99	356.99	x	x	x
B5	1328.97	663.98	442.32	x	x	x
B6	1649.95	824.47	549.31	x	x	x
Y0	102.09	50.54	33.36	x	x	x
Y1	423.07	211.03	140.35	x	x	x
Y2	679.06	339.03	225.68	x	x	x
Y3	1080.00	539.50	359.33	x	x	x
Y4	1256.03	627.51	418.01	x	x	x
Y5	1577.02	788.00	525.00	x	x	x
C1	193.03	96.01	63.67	x	x	x
C2	514.02	256.50	170.67	x	x	x
C3	690.05	344.52	229.34	x	x	x
C4	1090.99	544.99	362.99	x	x	x
C5	1346.98	672.98	448.32	x	x	x
C6	1667.96	833.48	555.31	x	x	x
Z0	84.08	41.54	27.36	x	x	x
Z1	405.06	202.03	134.35	x	x	x
Z2	661.05	330.02	219.68	x	x	x
Z3	1061.99	530.49	353.33	x	x	x
Z4	1238.02	618.51	412.00	x	x	x
Z5	1559.01	779.00	519.00	x	x	x

[1] Columns represent different charge states.

[2] Each CCS is an average of independent measurements with the corresponding standard deviation

Supplementary Table 26. CCS of B, Y, C and Z ions identified in a dp7 + 1x2*O*-sulfate, ΔUA-GlcNS-I2S-GlcNS-G-GlcNS-G-R_3 structure, where R_3 is $C_6H_4NO_2$.

**#36**								
ΔUA-GlcNS-I2S-GlcNS-G-GlcNS-G-R_3								
	(1-)[1]	(2-)	(3-)	(4-)	(1-)	(2-)	(3-)	(4-)
B1	157.01	78.00	51.67	38.50	70.5 (0.76)	x	x	x
B2	398.04	198.52	132.01	98.75	110.5 (0.8)	120.5 (0.5)	x	x
B3	654.03	326.51	217.34	162.75	x	162.6 (0.5)	x	x
B4	895.05	447.02	297.68	223.01	x	201.1 (0.5)	x	x
B5	1071.09	535.04	356.36	267.02	x	211.7 (0.3)	246.2 (0.8)	x
B6	1312.11	655.55	436.70	327.27	x	x	x	x
B7	1488.14	743.57	495.38	371.28	x	x	x	329.8 (0.1)
Y0	138.02	68.51	45.33	33.75	x	x	x	x
Y1	314.05	156.52	104.01	77.76	x	x	x	x
Y2	555.08	277.03	184.35	138.01	x	x	x	x
Y3	731.11	365.05	243.03	182.02	x	x	x	x
Y4	972.13	485.56	323.37	242.28	x	x	x	x
Y5	1228.12	613.56	408.70	306.27	x	x	x	x
Y6	1469.15	734.07	489.04	366.53	x	x	x	x
C1	174.02	86.50	57.33	42.75	x	x	x	x
C2	415.04	207.02	137.68	103.00	x	x	x	x
C3	671.03	335.01	223.01	167.00	x	x	x	x
C4	912.06	455.52	303.35	227.26	x	x	x	x
C5	1088.09	543.54	362.02	271.27	x	x	x	x
C6	1329.11	664.05	442.37	331.52	x	x	x	x
C7	1505.15	752.07	501.04	375.53	x	x	x	x
Z0	120.01	59.50	39.33	29.25	x	x	x	x

Z1	296.04	147.52	98.01	73.25	x	x	x	x
Z2	537.07	268.03	178.35	133.51	x	x	x	x
Z3	713.10	356.05	237.03	177.52	x	x	x	x
Z4	954.12	476.56	317.37	237.78	x	x	x	x
Z5	1210.11	604.55	402.70	301.77	x	x	x	x
Z6	1451.14	725.07	483.04	362.03	x	x	x	x

[1] Columns represent different charge states.

[2] Each CCS is an average of independent measurements with the corresponding standard deviation

Supplementary Table 27. CCS of B, Y, C and Z ($-SO_3$) ions identified in isomeric tetrasaccharide UA-GlcNAc6S-UA-GlcNAc6S-R_1 structures, where R_1 is $(CH_2)_5NH_2$.

			#9		#10		#11		#13	
			G-GlcNAc6S-G-GlcNAc6S-R_1		I-GlcNAc6S-I-GlcNAc6S-R_1		G-GlcNAc6S-I-GlcNAc6S-R_1		I-GlcNAc6S-G-GlcNAc6S-R_1	
			$- 1SO_3$	$- 1SO_3$	$- 1SO_3$	$- 1SO_3$	$- 1SO_3$	$- 1SO_3$	$- 1SO_3$	$- 2SO_3$
			(1-)[1]	(2-)	(1-)	(2-)	(1-)	(2-)	(1-)	(2-)
B1	95.07	47.03	x	x	x	x	x	x	x	x
B2	378.10	188.55	112.04 (0.1)	x	112.5 (0.3)	x	112.41 (0.5)	x	112.02 (0.2)	x
B3	554.14	276.56	x	x	x	x	x	x	x	x
B4	837.17	418.08	x	x	x	x	x	x	x	x
Y0	22.14	10.56	x	x	x	x	x	x	x	x
Y1	305.17	152.08	x	x	x	x	x	x	x	x
Y2	481.20	240.10	139.34 (0.2)	x	137.09 (0.3)	x	137.38 (0.6)	x	139.3 (0.2)	x
Y3	764.24	381.62	x	x	x	x		x	x	x
C1	113.08	56.04	x	x	x	x	x	x	x	x
C2	396.11	197.55	115.2 (0.6)	x	115.7 (0.5)	x	114.85 (0.4)	x	114.88 (0.2)	x
C3	572.15	285.57	x	x	x	x	x	x	x	x
C4	855.18	427.09	x	x	x	x	x	x	x	x
Z0	4.12	1.56	x	x	x	x	x	x	x	x
Z1	287.16	143.08	x	x	x	x	x	x	x	x
Z2	463.19	231.09	x	x	x	x	x	x	x	x
Z3	746.23	372.61	x	x	x	x	x	x	x	x

[1] Columns represent different charge states.

[2] Each CCS is an average of independent measurements with the corresponding standard deviation

Supplementary Table 28. CCS of B, Y, C and Z (-SO_3) ions identified in isomeric tetra-saccharide UA-GlcNS6S-UA-GlcNS6S-R_1 structures, where R_1 is $(CH_2)_5NH_2$.

**#12**						
G-GlcNS6S-I-GlcNS6S-R_1						
				- $1SO_3$	- $1SO_3$	- $1SO_3$
				(1-)[1]	(2-)	(3-)
B1	95.07	47.03	31.02	x	x	x
B2	416.05	207.52	138.01	x	122.4 (0.3)	x
B3	592.08	295.54	196.69	139.6 (0.3)	x	x
B4	913.06	456.03	303.68	x	x	214.9 (0.2)
Y0	22.14	10.56	6.71	x	x	x
Y1	343.12	171.05	113.70	x	x	x
Y2	519.15	259.07	172.38	x	x	x
Y3	840.13	419.56	279.37	x	x	x
C1	113.08	56.04	37.02	x	x	x
C2	434.06	216.53	144.01	x	x	x
C3	610.09	304.54	202.69	x	x	x
C4	931.07	465.03	309.69	x	x	x
Z0	4.12	1.56	0.70	x	x	x
Z1	325.11	162.05	107.70	x	x	x
Z2	501.14	250.07	166.37	x	x	x
Z3	822.12	410.56	273.37	x	x	x

[1] Columns represent different charge states.

[2] Each CCS is an average of independent measurements with the corresponding standard deviation

Supplementary Table 29. CCS of B, Y, C and Z (-SO_3) ions identified in isomeric tetra-saccharide UA-GlcNS6S-UA-GlcNS6S-R_1 structures, where R_1 is $(CH_2)_5NH_2$.

**#14**				
I-GlcNS6S-G-GlcNS6S-R_1				
	- $1SO_3$	- $1SO_3$	- $1SO_3$	- $1SO_3$
	(1-)	(2-)	(1-)	(2-)
B1	95.07	47.03	x	x
B2	416.05	207.52	x	x
B3	592.08	295.54	x	x
B4	913.06	456.03	x	x
Y0	22.14	10.56	x	x
Y1	343.12	171.05	108.9(0.1)[2]	x
Y2	519.15	259.07	133.7 (0.1)	149.8 (0.3)
Y3	840.13	419.56	x	175.7 (0.8)
C1	113.08	56.04	x	x
C2	434.06	216.53	x	x
C3	610.09	304.54	x	x
C4	931.07	465.03	x	x
Z0	4.12	1.56	x	x
Z1	325.11	162.05	x	x
Z2	501.14	250.07	x	x
Z3	822.12	410.56	x	x

[1] Columns represent different charge states.

[2] Each CCS is an average of independent measurements with the corresponding standard deviation

Supplementary Table 30. CCS of B, Y, C and Z (-SO_3) ions identified in isomeric tetra-saccharide G-GlcNS6S-I2S-GlcNS6S-R_1 structures, where R_1 is $(CH_2)_5NH_2$.

**#15**						
G-GlcNS6S-I2S-GlcNS6S-R_1						
	- 1SO_3	- 1SO_3	- 1SO_3			
	(1-)[1]	(2-)	(3-)			
B1	95.07	47.03	31.02	x	x	x
B2	416.05	207.52	138.01	x	122.4 (0.1)[2]	x
B3	672.04	335.52	223.34	x	167.1 (0.4)	x
B4	993.02	496.01	330.34	x	x	226.7 (0.2)
Y0	22.14	10.56	6.71	x	x	x
Y1	343.12	171.05	113.70	109.3 (0.6)	x	x
Y2	599.11	299.05	199.03	x	x	x
Y3	920.09	459.54	306.02	x	x	x
C1	113.08	56.04	37.02	x	x	x
C2	434.06	216.53	144.01	x	x	x
C3	690.05	344.52	229.34	x	x	x
C4	1011.03	505.01	336.34	x	x	x
Z0	4.12	1.56	0.70	x	x	x
Z1	325.11	162.05	107.70	x	x	x
Z2	581.10	290.04	193.03	x	x	x
Z3	902.08	450.54	300.02	x	x	x

[1] Columns represent different charge states.

[2] Each CCS is an average of independent measurements with the corresponding standard deviation

Supplementary Table 31. CCS of B, Y, C and Z (-SO_3) ions identified in a hexa-saccharide structure (R_1 is $(CH_2)_5NH_2$).

**#16**						
G-GlcNS6S-I-GlcNS6S-G-GlcNS6S-R_1						
	- 1SO_3	- 1SO_3	- 1SO_3	- 1SO_3	- 1SO_3	- 1SO_3
	(1-)[1]	(2-)	(3-)	(1-)	(2-)	(3-)
B1	95.07	47.03	31.02	x	x	x
B2	416.05	207.52	138.01	x	122.5 (0.2)[2]	x
B3	592.08	295.54	196.69	140.2 (0.7)	x	x
B4	913.06	456.03	303.68	x	x	214.8 (0.7)
B5	1089.10	544.04	362.36	x	x	247.4 (0.9)
B6	1410.08	704.54	469.35	x	x	x
Y0	22.14	10.56	6.71	x	x	x
Y1	343.12	171.05	113.70	109.1 (0.1)	x	x
Y2	519.15	259.07	172.38	x	150.0 (0.8)	x
Y3	840.13	419.56	279.37	x	x	x
Y4	1016.16	507.58	338.05	x	x	234.8 (0.2)
Y5	1337.15	668.07	445.04	x	x	x
C1	113.08	56.04	37.02	x	x	x
C2	434.06	216.53	144.01	114.8 (0.46)	x	x
C3	610.09	304.54	202.69	x	x	x
C4	931.07	465.03	309.69	x	x	x
C5	1107.11	553.05	368.36	x	x	x
C6	1428.09	713.54	475.36	x	x	x
Z0	4.12	1.56	0.70	x	x	x
Z1	325.11	162.05	107.70	x	x	x
Z2	501.14	250.07	166.37	x	x	x
Z3	822.12	410.56	273.37	x	x	x
Z4	998.15	498.57	332.05	x	x	x
Z5	1319.14	659.06	439.04	x	x	x

[1] Columns represent different charge states.

[2] Each CCS is an average of independent measurements with the corresponding standard deviation

Supplementary Table 32. CCS of B, Y, C and Z (-SO_3) ions identified in a hexa-saccharide GlcNAc6S-[G-GlcNAc6S]$_2$-G-R_2 structure, where R_2 is C_7H_7O.

**#17**						
GlcNAc6S-[G-GlcNAc6S]$_2$-G-R_2						
	- 1SO_3	- 1SO_3	- 1SO_3	- 1SO_3	- 1SO_3	- 1SO_3
	(1-)[1]	(2-)	(3-)	(1-)	(2-)	(3-)
B1	202.07	100.53	66.69	x	x	x
B2	378.10	188.55	125.36	111.4 (0.2)	x	x
B3	661.14	330.07	219.71	160.1 (0.7)	x	x
B4	837.17	418.08	278.39	x	x	x
B5	1120.21	559.60	372.73	x	214.1 (0.7)	x
B6	1296.24	647.62	431.41	x	234.0 (0.4)	x
Y0	42.08	20.54	13.35	x	x	x
Y1	219.12	109.06	72.37	x	x	x
Y2	502.16	250.57	166.71	x	x	x
Y3	678.19	338.59	225.39	x	x	x
Y4	961.22	480.11	319.74	x	x	x
Y5	1137.26	568.12	378.41	x	x	x
C1	220.08	109.54	72.69	x	x	x
C2	396.11	197.55	131.37	x	x	x
C3	679.15	339.07	225.71	x	x	x
C4	855.18	427.09	284.39	x	x	x
C5	1138.22	568.61	378.73	x	x	x
C6	1314.25	656.62	437.41	x	x	x
Z0	25.08	12.03	7.69	x	x	x
Z1	201.11	100.05	66.36	x	x	x
Z2	484.15	241.57	160.71	x	x	x
Z3	660.18	329.58	219.39	x	x	x
Z4	943.21	471.10	313.73	x	x	x
Z5	1119.25	559.12	372.41	x	x	X

[1] Columns represent different charge states.

[2] Each CCS is an average of independent measurements with the corresponding standard deviation

Supplementary Table 33. CCS of B, Y, C and Z (-SO_3) ions identified in a hexa-saccharide GlcNAc6S-[I-GlcNAc6S]$_2$-I-R_2 structure, where R_2 is C_7H_7O.

**#18**						
GlcNAc6S-[I-GlcNAc6S]$_2$-I-R_2						
	- 1SO_3	- 1SO_3	- 1SO_3	- 1SO_3	- 1SO_3	- 1SO_3
	(1-)[1]	(2-)	(3-)	(1-)	(2-)	(3-)
B1	202.07	100.53	66.69	x	x	x
B2	378.10	188.55	125.36	112.5 (0.1)	x	x
B3	661.14	330.07	219.71	157.7 (0.3)	x	x
B4	837.17	418.08	278.39	x	179.8 (0.9)	x
B5	1120.21	559.60	372.73	x	215.6 (0.2)	x
B6	1296.24	647.62	431.41	x	237.8 (0.4)	x
Y0	42.08	20.54	13.35	x	x	x
Y1	219.12	109.06	72.37	x	x	x
Y2	502.16	250.57	166.71	x	x	x
Y3	678.19	338.59	225.39	x	x	x
Y4	961.22	480.11	319.74	x	x	x
Y5	1137.26	568.12	378.41	x	x	x
C1	220.08	109.54	72.69	x	x	x
C2	396.11	197.55	131.37	x	x	x
C3	679.15	339.07	225.71	x	x	x
C4	855.18	427.09	284.39	x	x	x
C5	1138.22	568.61	378.73	x	x	x
C6	1314.25	656.62	437.41	x	x	x
Z0	25.08	12.03	7.69	x	x	x
Z1	201.11	100.05	66.36	x	x	x
Z2	484.15	241.57	160.71	x	x	x
Z3	660.18	329.58	219.39	x	x	x
Z4	943.21	471.10	313.73	x	x	x
Z5	1119.25	559.12	372.41	x	x	x

[1] Columns represent different charge states.

[2] Each CCS is an average of independent measurements with the corresponding standard deviation

Supplementary Table 34. CCS of B, Y, C and Z (-SO_3) ions identified in an octa-saccharide GlcNAc6S-[I-GlcNAc6S]$_3$-I-R_2 structure, where R_2 is C_7H_7O.

**#19**						
GlcNAc6S-[I-GlcNAc6S]$_3$-I-R_2						
	- 1SO_3	- 1SO_3	- 1SO_3	- 1SO_3	- 1SO_3	- 1SO_3
	(1-)[1]	(2-)	(3-)	(1-)	(2-)	(3-)
B1	202.07	100.53	66.69	x	x	x
B2	378.10	188.55	125.36	112.0 (0.3)[2]	x	x
B3	661.14	330.07	219.71	158.2 (0.2)	x	x
B4	837.17	418.08	278.39	x	x	x
B5	1120.21	559.60	372.73	x	216.3 (0.7)	x
B6	1296.24	647.62	431.41	x	x	x
B7	1579.28	789.13	525.75	x	x	x
B8	1755.31	877.15	584.43	x	x	
Y0	42.08	20.54	13.35	x	x	x
Y1	219.12	109.06	72.37	x	x	x
Y2	502.16	250.57	166.71	x	x	x
Y3	678.19	338.59	225.39	x	x	x
Y4	961.22	480.11	319.74	x	x	x
Y5	1137.26	568.12	378.41	x	x	x
Y6	1420.29	709.64	472.76	x	x	x
Y7	1596.32	797.66	531.44	x	x	x
C1	220.08	109.54	72.69	x	x	x
C2	396.11	197.55	131.37	x	x	x
C3	679.15	339.07	225.71	x	x	x
C4	855.18	427.09	284.39	x	x	x
C5	1138.22	568.61	378.73	x	x	x
C6	1314.25	656.62	437.41	x	x	x
C7	1597.29	798.14	531.76	x	x	x
C8	1773.32	886.16	590.43	x	x	x
Z0	25.08	12.03	7.69	x	x	x
Z1	201.11	100.05	66.36	x	x	x
Z2	484.15	241.57	160.71	x	x	x
Z3	660.18	329.58	219.39	x	x	x
Z4	943.21	471.10	313.73	x	x	x
Z5	1119.25	559.12	372.41	x	x	x
Z6	1402.28	700.64	466.76	x	x	x
Z7	1578.31	788.65	525.43	x	x	x

[1] Columns represent different charge states. [2] Each CCS is an average of independent measurements with the corresponding standard deviation

Supplementary Table 35. CCS of B, Y, C and Z (-SO_3) ions identified in an octa-saccharide GlcNAc6S-[G-GlcNAc6S]$_3$-G-R_2 structure, where R_2 is C_7H_7O.

**#20**						
GlcNAc6S-[G-GlcNAc6S]$_3$-G-R_2						
	- 1SO_3	- 1SO_3	- 1SO_3	- 1SO_3	- 1SO_3	- 1SO_3
	(1-)[1]	(2-)	(3-)	(1-)	(2-)	(3-)
B1	202.07	100.53	66.69	x	x	x
B2	378.10	188.55	125.36	111.5 (0.1)[2]	x	x
B3	661.14	330.07	219.71	160.4 (0.2)	x	x
B4	837.17	418.08	278.39	x	185.4 (0.4)	x
B5	1120.21	559.60	372.73	x	214.9 (0.8)	x
B6	1296.24	647.62	431.41	x	x	x
B7	1579.28	789.13	525.75	x	x	x
B8	1755.31	877.15	584.43	x	x	x
Y0	42.08	20.54	13.35	x	x	x
Y1	219.12	109.06	72.37	x	x	x
Y2	502.16	250.57	166.71	x	x	x
Y3	678.19	338.59	225.39	x	x	x
Y4	961.22	480.11	319.74	x	199.7 (0.18)	x
Y5	1137.26	568.12	378.41	x	216.2 (0.1)	x
Y6	1420.29	709.64	472.76	x	x	x
Y7	1596.32	797.66	531.44	x	x	x
C1	220.08	109.54	72.69	x	x	x
C2	396.11	197.55	131.37	x	x	x
C3	679.15	339.07	225.71	x	x	x
C4	855.18	427.09	284.39	x	x	x
C5	1138.22	568.61	378.73	x	x	x
C6	1314.25	656.62	437.41	x	x	x
C7	1597.29	798.14	531.76	x	x	x
C8	1773.32	886.16	590.43	x	x	x
Z0	25.08	12.03	7.69	x	x	x
Z1	201.11	100.05	66.36	x	x	x
Z2	484.15	241.57	160.71	x	x	x
Z3	660.18	329.58	219.39	x	x	x
Z4	943.21	471.10	313.73	x	x	x
Z5	1119.25	559.12	372.41	x	x	x
Z6	1402.28	700.64	466.76	x	x	x
Z7	1578.31	788.65	525.43	x	x	x

[1] Columns represent different charge states.

[2] Each CCS is an average of independent measurements with the corresponding standard deviation

Supplementary Table 36. CCS of B, Y, C and Z ($-SO_3$) ions identified in an octa-saccharide GlcNAc6S-G-GlcNac6S-G-GlcNAc6S-I-GlcNAc6S-I-R_2 structure, where R_2 is C_7H_7O.

#21						
GlcNAc6S-G-GlcNac6S-G-GlcNAc6S-I-GlcNAc6S-I-R_2						
	- $1SO_3$	- $1SO_3$	- $1SO_3$	- $1SO_3$	- $1SO_3$	- $1SO_3$
	(1-)[1]	(2-)	(3-)	(1-)[1]	(2-)	(3-)
B1	202.07	100.53	66.69	x	x	x
B2	378.10	188.55	125.36	112.3 (0.3)[2]	x	x
B3	661.14	330.07	219.71	x	x	x
B4	837.17	418.08	278.39	x	x	x
B5	1120.21	559.60	372.73	x	213.8 (0.1)	x
B6	1296.24	647.62	431.41	x	x	x
B7	1579.28	789.13	525.75	x	x	x
B8	1755.31	877.15	584.43	x	x	x
Y0	42.08	20.54	13.35	x	x	x
Y1	219.12	109.06	72.37	x	x	x
Y2	502.16	250.57	166.71	x	x	x
Y3	678.19	338.59	225.39	x	x	x
Y4	961.22	480.11	319.74	x	x	x
Y5	1137.26	568.12	378.41	x	x	x
Y6	1420.29	709.64	472.76	x	x	x
Y7	1596.32	797.66	531.44	x	x	x
C1	220.08	109.54	72.69	x	x	x
C2	396.11	197.55	131.37	x	x	x
C3	679.15	339.07	225.71	x	x	x
C4	855.18	427.09	284.39	x	x	x
C5	1138.22	568.61	378.73	x	x	x
C6	1314.25	656.62	437.41	x	x	x
C7	1597.29	798.14	531.76	x	x	x
C8	1773.32	886.16	590.43	x	x	x
Z0	25.08	12.03	7.69	x	x	x
Z1	201.11	100.05	66.36	x	x	x
Z2	484.15	241.57	160.71	x	x	x
Z3	660.18	329.58	219.39	x	x	x
Z4	943.21	471.10	313.73	x	x	x
Z5	1119.25	559.12	372.41	x	x	x
Z6	1402.28	700.64	466.76	x	x	x
Z7	1578.31	788.65	525.43	x	x	x

[1] Columns represent different charge states.

[2] Each CCS is an average of independent measurements with the corresponding standard deviation

Supplementary Table 37. CCS of B, Y, C and Z (-SO_3) ions identified in an octa-saccharide GlcNAc6S-G-GlcNac6S-I-GlcNAc6S-I-GlcNAc6S-G-R_2 structure, where R_2 is C_7H_7O.

**#22**						
GlcNAc6S-G-GlcNac6S-I-GlcNAc6S-I-GlcNAc6S-G-R_2						
	- 1SO_3	- 1SO_3	- 1SO_3	- 1SO_3	- 1SO_3	- 1SO_3
	(1-)[1]	(2-)	(3-)	(1-)[1]	(2-)	(3-)
B1	202.07	100.53	66.69	x	x	x
B2	378.10	188.55	125.36	112.9 (0.6)[2]	x	x
B3	661.14	330.07	219.71	159.6 (0.5)	x	x
B4	837.17	418.08	278.39	x	x	x
B5	1120.21	559.60	372.73	x	216.3 (0.33)	x
B6	1296.24	647.62	431.41	x	x	x
B7	1579.28	789.13	525.75	x	x	x
B8	1755.31	877.15	584.43	x	x	308.6 (0.5)
Y0	42.08	20.54	13.35	x	x	x
Y1	219.12	109.06	72.37	x	x	x
Y2	502.16	250.57	166.71	x	x	x
Y3	678.19	338.59	225.39	x	x	x
Y4	961.22	480.11	319.74	x	x	x
Y5	1137.26	568.12	378.41	x	x	x
Y6	1420.29	709.64	472.76	x	x	x
Y7	1596.32	797.66	531.44	x	x	x
C1	220.08	109.54	72.69	x	x	x
C2	396.11	197.55	131.37	x	x	x
C3	679.15	339.07	225.71	x	x	x
C4	855.18	427.09	284.39	x	x	x
C5	1138.22	568.61	378.73	x	x	x
C6	1314.25	656.62	437.41	x	x	x
C7	1597.29	798.14	531.76	x	x	x
C8	1773.32	886.16	590.43	x	x	x
Z0	25.08	12.03	7.69	x	x	x
Z1	201.11	100.05	66.36	x	x	x
Z2	484.15	241.57	160.71	x	x	x
Z3	660.18	329.58	219.39	x	x	x
Z4	943.21	471.10	313.73	x	x	x
Z5	1119.25	559.12	372.41	x	x	x
Z6	1402.28	700.64	466.76	x	x	x
Z7	1578.31	788.65	525.43	x	x	x

[1] Columns represent different charge states.

[2] Each CCS is an average of independent measurements with the corresponding standard deviation

Supplementary Table 38. CCS of B, Y, C and Z (-SO_3) ions identified in an octa-saccharide GlcNAc6S-I-GlcNAc6S-G-GlcNAc6S-I-GlcNAc6S-G-R_2 structure, where R_2 is C_7H_7O.

**#23**						
GlcNAc6S-I-GlcNAc6S-G-GlcNAc6S-I-GlcNAc6S-G-R_2						
	- 1SO_3	- 1SO_3	- 1SO_3	- 1SO_3	- 1SO_3	- 1SO_3
	(1-)[1]	(2-)	(3-)	(1-)[1]	(2-)	(3-)
B1	202.07	100.53	66.69	x	x	x
B2	378.10	188.55	125.36	112.1 (0.3)[2]	x	x
B3	661.14	330.07	219.71	157.8 (0.2)	x	x
B4	837.17	418.08	278.39	x	x	x
B5	1120.21	559.60	372.73	x	215.1 (0.11)	x
B6	1296.24	647.62	431.41	x	x	x
B7	1579.28	789.13	525.75	x	x	x
B8	1755.31	877.15	584.43	x	x	x
Y0	42.08	20.54	13.35	x	x	x
Y1	219.12	109.06	72.37	x	x	x
Y2	502.16	250.57	166.71	x	x	x
Y3	678.19	338.59	225.39	x	x	x
Y4	961.22	480.11	319.74	x	x	x
Y5	1137.26	568.12	378.41	x	x	x
Y6	1420.29	709.64	472.76	x	x	x
Y7	1596.32	797.66	531.44	x	x	x
C1	220.08	109.54	72.69	x	x	x
C2	396.11	197.55	131.37	x	x	x
C3	679.15	339.07	225.71	x	x	x
C4	855.18	427.09	284.39	x	x	x
C5	1138.22	568.61	378.73	x	x	x
C6	1314.25	656.62	437.41	x	x	x
C7	1597.29	798.14	531.76	x	x	x
C8	1773.32	886.16	590.43	x	x	x
Z0	25.08	12.03	7.69	x	x	x
Z1	201.11	100.05	66.36	x	x	x
Z2	484.15	241.57	160.71	x	x	x
Z3	660.18	329.58	219.39	x	x	x
Z4	943.21	471.10	313.73	x	x	x
Z5	1119.25	559.12	372.41	x	x	x
Z6	1402.28	700.64	466.76	x	x	x
Z7	1578.31	788.65	525.43	x	x	x

[1] Columns represent different charge states.

[2] Each CCS is an average of independent measurements with the corresponding standard deviation

Supplementary Table 39. CCS of B, Y, C and Z (-SO_3) ions identified in an octa-saccharide GlcNAc6S-G-GlcNAc6S-I-GlcNAc6S-G-GlcNAc6S-I-R_2 structure, where R_2 is C_7H_7O.

**#24**						
GlcNAc6S-G-GlcNAc6S-I-GlcNAc6S-G-GlcNAc6S-I-R_2						
	- $1SO_3$	- $1SO_3$	- $1SO_3$	- $1SO_3$	- $1SO_3$	- $1SO_3$
	(1-)[1]	(2-)	(3-)	(1-)	(2-)	(3-)
B1	202.07	100.53	66.69	x	x	x
B2	378.10	188.55	125.36	111.64 (0.2)	x	x
B3	661.14	330.07	219.71	x	x	x
B4	837.17	418.08	278.39	x	x	x
B5	1120.21	559.60	372.73	x	215.7 (0.6)	x
B6	1296.24	647.62	431.41	x	x	x
B7	1579.28	789.13	525.75	x	x	x
B8	1755.31	877.15	584.43	x	x	x
Y0	42.08	20.54	13.35	x	x	x
Y1	219.12	109.06	72.37	x	x	x
Y2	502.16	250.57	166.71	x	x	x
Y3	678.19	338.59	225.39	x	x	x
Y4	961.22	480.11	319.74	x	x	x
Y5	1137.26	568.12	378.41	x	x	x
Y6	1420.29	709.64	472.76	x	x	x
Y7	1596.32	797.66	531.44	x	x	x
C1	220.08	109.54	72.69	x	x	x
C2	396.11	197.55	131.37	x	x	x
C3	679.15	339.07	225.71	x	x	x
C4	855.18	427.09	284.39	x	x	x
C5	1138.22	568.61	378.73	x	x	x
C6	1314.25	656.62	437.41	x	x	x
C7	1597.29	798.14	531.76	x	x	x
C8	1773.32	886.16	590.43	x	x	x
Z0	25.08	12.03	7.69	x	x	x
Z1	201.11	100.05	66.36	x	x	x
Z2	484.15	241.57	160.71	x	x	x
Z3	660.18	329.58	219.39	x	x	x
Z4	943.21	471.10	313.73	x	x	x
Z5	1119.25	559.12	372.41	x	x	x
Z6	1402.28	700.64	466.76	x	x	x
Z7	1578.31	788.65	525.43	x	x	x

[1] Columns represent different charge states.

[2] Each CCS is an average of independent measurements with the corresponding standard deviation

Supplementary Table 40. CCS of B, Y, C and Z (-SO_3) ions identified in a 9mer, G-GlcNS-G-GlcNS-I-GlcNS-G-GlcNS-G-R_3 structure, where R_3 is $C_6H_4NO_2$.

**#25**						
G-GlcNS-G-GlcNS-I-GlcNS-G-GlcNS-G-R_3						
	- 1SO_3	- 1SO_3	- 1SO_3			
	(1-)[1]	(2-)	(3-)	(1-)	(2-)	(3-)
B1	95.07	47.03	31.02	x	x	x
B2	336.09	167.54	111.36	x	x	x
B3	512.13	255.56	170.04	132.30 (0.5)[2]	x	x
B4	753.15	376.07	250.38	x	x	x
B5	929.18	464.09	309.06	x	x	x
B6	1170.21	584.60	389.40	x	227.58 (0.2)	x
B7	1346.24	672.62	448.07	x	x	x
B8	1587.27	793.13	528.42	x	x	x
B9	1763.30	881.15	587.09	x	x	x
Y0	58.06	28.53	18.68	x	x	x
Y1	234.09	116.54	77.36	x	x	x
Y2	475.12	237.06	157.70	x	x	x
Y3	651.15	325.07	216.38	x	x	x
Y4	892.18	445.58	296.72	x	x	x
Y5	1068.21	533.60	355.40	x	x	x
Y6	1309.24	654.11	435.74	x	x	x
Y7	1485.27	742.13	494.42	x	x	x
Y8	1726.29	862.64	574.76	x	x	x
C1	112.07	55.53	36.68	x	x	x
C2	353.10	176.04	117.03	x	x	x
C3	529.13	264.06	175.70	x	x	x
C4	770.15	384.57	256.05	x	x	x
C5	946.19	472.59	314.72	x	x	x
C6	1187.21	593.10	395.07	x	x	x
C7	1363.24	681.12	453.74	x	x	x
C8	1604.27	801.63	534.08	x	x	x
C9	1780.30	889.65	592.76	x	x	x
Z0	40.05	19.52	12.68	x	x	x
Z1	216.08	107.54	71.36	x	x	x
Z2	457.11	228.05	151.70	x	x	x
Z3	633.14	316.07	210.38	x	x	x
Z4	874.17	436.58	290.72	x	x	x
Z5	1050.20	524.60	349.39	x	x	x
Z6	1291.22	645.11	429.74	x	x	x
Z7	1467.26	733.12	488.41	x	x	x

Z8	1708.28	853.64	568.76	x	x	x

[1] Columns represent different charge states.

[2] Each CCS is an average of independent measurements with the corresponding standard deviation

Supplementary Table 41. CCS of B, Y, C and Z (-SO_3) ions identified in a dp9 + 1x2*O*-sulfate, G-GlcNS-G-GlcNS-I2S-GlcNS-G-GlcNS-G-R_3 structure, where R_3 is $C_6H_4NO_2$.

**#26**						
G-GlcNS-G-GlcNS-I2S-GlcNS-G-GlcNS-G-R_3						
	- 1SO_3	- 1SO_3	- 1SO_3	- 1SO_3	- 1SO_3	- 1SO_3
	(1-)[1]	(2-)	(3-)	(1-)	(2-)	(3-)
B1	95.07	47.03	31.02	x	x	x
B2	336.09	167.54	111.36	x	x	x
B3	512.13	255.56	170.04	131.6 (0.5)[2]	x	x
B4	753.15	376.07	250.38	x	x	x
B5	1009.14	504.07	335.71	x	234.21 (0.2)	x
B6	1250.17	624.58	416.05	x	x	x
B7	1426.20	712.59	474.73	x	x	x
B8	1667.22	833.11	555.07	x	x	x
B9	1843.25	921.12	613.75	x	x	x
Y0	58.06	28.53	18.68	x	x	x
Y1	234.09	116.54	77.36	x	x	x
Y2	475.12	237.06	157.70	x	x	x
Y3	651.15	325.07	216.38	x	x	x
Y4	892.18	445.58	296.72	x	x	x
Y5	1148.17	573.58	382.05	x	x	x
Y6	1389.19	694.09	462.39	x	x	x
Y7	1565.22	782.11	521.07	x	x	x
Y8	1806.25	902.62	601.41	x	x	x
C1	112.07	55.53	36.68	x	x	x
C2	353.10	176.04	117.03	x	x	x
C3	529.13	264.06	175.70	x	x	x
C4	770.15	384.57	256.05	x	x	x
C5	1026.14	512.57	341.38	x	x	x
C6	1267.17	633.08	421.72	x	x	x
C7	1443.20	721.10	480.39	x	x	x
C8	1684.23	841.61	560.74	x	x	x
C9	1860.26	929.62	619.41	x	x	x
Z0	40.05	19.52	12.68	x	x	x
Z1	216.08	107.54	71.36	x	x	x
Z2	457.11	228.05	151.70	x	x	x
Z3	633.14	316.07	210.38	x	x	x
Z4	874.17	436.58	290.72	x	x	x
Z5	1130.16	564.57	376.05	x	x	x
Z6	1371.18	685.09	456.39	x	x	x
Z7	1547.21	773.10	515.07	x	x	x

Z8	1788.24	893.62	595.41	x	x	x

[1] Columns represent different charge states.

[2] Each CCS is an average of independent measurements with the corresponding standard deviation

Supplementary Table 42. CCS of B, Y, C and Z (-SO_3) ions identified in a dp10 GlcNAc6S-[I-GlcNAc6S]$_4$-I-R_2 structure, where R_2 is C_7H_7O.

**#27**								
GlcNAc6S-[I-GlcNAc6S]$_4$-I-R_2								
	- 1SO_3	- 1SO_3	- 1SO_3	- 1SO_3	- 1SO_3	- 1SO_3	- 1SO_3	- 1SO_3
	(1-)[1]	(2-)	(3-)	(4-)	(1-)	(2-)	(3-)	(4-)
B1	202.07	100.53	66.69	49.76	x	x	x	x
B2	378.10	188.55	125.36	93.77	112.38 (0.2)[2]	x	x	x
B3	661.14	330.07	219.71	164.53	x	167.6 (0.5)	x	x
B4	837.17	418.08	278.39	208.54	x	x	x	x
B5	1120.21	559.60	372.73	279.30	x	215.7 (0.2)	255.5 (0.4)	x
B6	1296.24	647.62	431.41	323.30	x	x	283.0 (0.5)	x
B7	1579.28	789.13	525.75	394.06	x	x	x	x
B8	1755.31	877.15	584.43	438.07	x	x	x	x
B9	2038.34	1018.67	678.78	508.83	x	x	x	x
B10	2214.38	1106.68	737.45	552.84	x	x	x	x
Y0	43.09	21.04	13.69	10.02	x	x	x	x
Y1	219.12	109.06	72.37	54.02	x	x	x	x
Y2	502.16	250.57	166.71	124.78	x	x	x	x
Y3	678.19	338.59	225.39	168.79	x	x	x	x
Y4	961.22	480.11	319.74	239.55	x	x	x	x
Y5	1137.26	568.12	378.41	283.56	x	x	x	x
Y6	1420.29	709.64	472.76	354.32	x	x	x	x
Y7	1596.32	797.66	531.44	398.33	x	x	x	x
Y8	1879.36	939.18	625.78	469.08	x	x	x	x
Y9	2055.39	1027.19	684.46	513.09	x	x	x	x
C1	220.08	109.54	72.69	54.26	x	x	x	x
C2	396.11	197.55	131.37	98.27	x	x	x	x
C3	679.15	339.07	225.71	169.03	x	x	x	x
C4	855.18	427.09	284.39	213.04	x	x	x	x
C5	1138.22	568.61	378.73	283.80	x	x	x	x

C6	1314.25	656.62	437.41	327.81	x	x	x	x
C7	1597.29	798.14	531.76	398.57	x	x	x	x
C8	1773.32	886.16	590.43	442.57	x	x	x	x
C9	2056.35	1027.67	684.78	513.33	x	x	x	x
C10	2232.39	1115.69	743.46	557.34	x	x	x	x
Z0	25.08	12.03	7.69	5.51	x	x	x	x
Z1	201.11	100.05	66.36	49.52	x	x	x	x
Z2	484.15	241.57	160.71	120.28	x	x	x	x
Z3	660.18	329.58	219.39	164.29	x	x	x	x
Z4	943.21	471.10	313.73	235.05	x	x	x	x
Z5	1119.25	559.12	372.41	279.06	x	x	x	x
Z6	1402.28	700.64	466.76	349.81	x	x	x	x
Z7	1578.31	788.65	525.43	393.82	x	x	x	x
Z8	1861.35	930.17	619.78	464.58	x	x	x	x
Z9	2037.38	1018.19	678.46	508.59	x	x	x	x

[1] Columns represent different charge states.

[2] Each CCS is an average of independent measurements with the corresponding standard deviation

Supplementary Table 43. CCS of B, Y, C and Z (-SO_3) ions identified in a dp10 GlcNAc6S-[G-GlcNAc6S]$_4$-G-R_2 structure, where R_2 is C_7H_7O.

**#28**								
GlcNAc6S-[G-GlcNAc6S]4-G-R_2								
	(1-)[1]	(2-)	(3-)	(4-)	(1-)	(2-)	(3-)	(4-)
B1	202.07	100.53	66.69	49.76	x	x	x	x
B2	378.10	188.55	125.36	93.77	111.3 (0.2)[2]	x	x	x
B3	661.14	330.07	219.71	164.53	159.9 (0.2)	x	x	x
B4	837.17	418.08	278.39	208.54	x	x	x	x
B5	1120.21	559.60	372.73	279.30	x	214.4 (0.8)	x	x
B6	1296.24	647.62	431.41	323.30	x	x	x	x
B7	1579.28	789.13	525.75	394.06	x	x	300.8 (0.5)	x
B8	1755.31	877.15	584.43	438.07	x	x	x	x
B9	2038.34	1018.67	678.78	508.83	x	x	x	x
B10	2214.38	1106.68	737.45	552.84	x	x	x	x
Y0	43.09	21.04	13.69	10.02	x	x	x	x
Y1	219.12	109.06	72.37	54.02	x	x	x	x
Y2	502.16	250.57	166.71	124.78	x	x	x	x
Y3	678.19	338.59	225.39	168.79	x	x	x	x
Y4	961.22	480.11	319.74	239.55	x	x	x	x
Y5	1137.26	568.12	378.41	283.56	x	x	x	x
Y6	1420.29	709.64	472.76	354.32	x	x	x	x
Y7	1596.32	797.66	531.44	398.33	x	x	x	x
Y8	1879.36	939.18	625.78	469.08	x	x	x	x
Y9	2055.39	1027.19	684.46	513.09	x	x	x	x
C1	220.08	109.54	72.69	54.26	x	x	x	x
C2	396.11	197.55	131.37	98.27	x	x	x	x
C3	679.15	339.07	225.71	169.03	x	x	x	x
C4	855.18	427.09	284.39	213.04	x	x	x	x
C5	1138.22	568.61	378.73	283.80	x	x	x	x

C6	1314.25	656.62	437.41	327.81	x	x	x	x
C7	1597.29	798.14	531.76	398.57	x	x	x	x
C8	1773.32	886.16	590.43	442.57	x	x	x	x
C9	2056.35	1027.67	684.78	513.33	x	x	x	x
C10	2232.39	1115.69	743.46	557.34	x	x	x	x
Z0	25.08	12.03	7.69	5.51	x	x	x	x
Z1	201.11	100.05	66.36	49.52	x	x	x	x
Z2	484.15	241.57	160.71	120.28	x	x	x	x
Z3	660.18	329.58	219.39	164.29	x	x	x	x
Z4	943.21	471.10	313.73	235.05	x	x	x	x
Z5	1119.25	559.12	372.41	279.06	x	x	x	x
Z6	1402.28	700.64	466.76	349.81	x	x	x	x
Z7	1578.31	788.65	525.43	393.82	x	x	x	x
Z8	1861.35	930.17	619.78	464.58	x	x	x	x
Z9	2037.38	1018.19	678.46	508.59	x	x	x	x

[1] Columns represent different charge states.

[2] Each CCS is an average of independent measurements with the corresponding standard deviation

Supplementary Table 44. CCS of B, Y, C and Z $-SO_3$ ions identified in disaccharide ΔUA2S-GlcNS3S6S structure.

**#29**				
ΔUA2S-GlcNS3S6S				
	- $1SO_3$	- $1SO_3$	- $1SO_3$	- $1SO_3$
	(1-)[1]	(2-)	(1-)	(2-)
B1	157.01	78.00	69.4 (0.1)[2]	x
Y0	337.99	168.49	x	106 (0.5)
C1	175.02	87.01	x	x
Z0	319.97	159.48	x	x

[1] Columns represent different charge states.

[2] Each CCS is an average of independent measurements with the corresponding standard deviation

Supplementary Table 45. CCS of B, Y, C and Z $-SO_3$ ions identified in tetraaccharide ΔUA-GlcNS-IdoA2S-GlcNS3S structure.

**#30**				
ΔUA-GlcNS-IdoA2S-GlcNS3S				
	$- 1SO_3$	$- 1SO_3$	$- 1SO_3$	$- 1SO_3$
	(1-)[1]	(2-)	(1-)	(2-)
B1	77.06	38.02	x	x
B2	318.08	158.54	99.5 (0.5)[2]	x
B3	574.07	286.53	141.4 (0.1)	150.5 (0.1)
Y0	258.03	128.51	85.3 (0.1)	x
Y1	514.02	256.50	x	133.0 (0.5)
Y2	755.04	377.02	x	172.8 (0.1)
C1	95.07	47.03	x	x
C2	336.09	167.54	x	x
C3	592.08	295.54	x	148.5 (0.4)

[1] Columns represent different charge states.

[2] Each CCS is an average of independent measurements with the corresponding standard deviation

Supplementary Table 46. CCS of B, Y, C and Z $-SO_3$ ions identified in tetrasaccharide ΔUA-GlcNS6S-GlcA-GlcNS3S6S structure.

#31				
UA-GlcNS6S-G-GlcNS3S6S				
	- $1SO_3$	- $1SO_3$	- $1SO_3$	- $1SO_3$
	(1-)[1]	(2-)	(1-)	(2-)
B1	77.06	38.02	x	x
B2	398.04	198.52	x	x
B3	574.07	286.53	140.9 (0.5)[2]	151.1 (0.3)
Y0	337.99	168.49	x	105.8 (0.3)
Y1	514.02	256.50	x	133.5 (0.5)
Y2	835.00	417.00	x	x
C1	95.07	47.03	x	x
C2	416.05	207.52	x	x
C3	592.08	295.54	133.5 (0.5)	x
Z0	319.97	159.48	x	x
Z1	496.01	247.50	x	x
Z2	816.99	407.99	x	x

[1] Columns represent different charge states.

[2] Each CCS is an average of independent measurements with the corresponding standard deviation

Supplementary Table 47. CCS of B, Y, C and Z $-SO_3$ ions identified in tetrasaccharide ΔUA-GlcNAc6S-GlcA-GlcNS3S6S structure.

**#32**				
ΔUA-GlcNAc6S-GlcA-GlcNS3S6S				
	$- 1SO_3$	$- 1SO_3$	$- 1SO_3$	$- 1SO_3$
	(1-)[1]	(2-)	(1-)	(2-)
B1	77.06	38.02	x	x
B2	360.09	179.54	x	x
B3	536.13	267.56	136.7 (0.2)[2]	x
Y0	337.99	168.49	x	x
Y1	514.02	256.50	x	x
Y2	797.05	398.02	x	x
C1	95.07	47.03	x	x
C2	378.10	188.55	x	x
C3	554.14	276.56	138.13 (0.2)	x
Z0	319.97	159.48	x	x
Z1	496.01	247.50	x	x
Z2	779.04	389.02	x	x

[1] Columns represent different charge states.

[2] Each CCS is an average of independent measurements with the corresponding standard deviation

Supplementary Table 48. CCS of B, Y, C and Z ($-SO_3$) ions identified in a hexasaccharide G-GlcNS6S-G-GlcNS6S-I2S-GlcNS6S-R_1 structure, where R_1 is $(CH_2)_5NH_2$.

**#33**						
G-GlcNS6S-G-GlcNS6S-I2S-GlcNS6S-R_1						
	- $1SO_3$	- $1SO_3$	- $1SO_3$	- $1SO_3$	- $1SO_3$	- $1SO_3$
	(1-)[1]	(2-)	(3-)	(1-)	(2-)	(3-)
B1	95.07	47.03	31.02	x	x	x
B2	416.05	207.52	138.01	x	122.2 (0.6)[2]	x
B3	592.08	295.54	196.69	x	152.5 (0.2)	x
B4	913.06	456.03	303.68	x	x	216.5 (0.3)
B5	1169.05	584.02	389.01	x	x	x
B6	1490.04	744.51	496.01	x	x	x
Y0	22.14	10.56	6.71	x	x	x
Y1	343.12	171.05	113.70	109.1 (0.3)	x	x
Y2	599.11	299.05	199.03	x	157.0 (0.2)	x
Y3	920.09	459.54	306.02	x	x	x
Y4	1096.12	547.56	364.70	x	x	246.3 (0.6)
Y5	1417.10	708.05	471.70	x	x	x
C1	113.08	56.04	37.02	x	x	x
C2	434.06	216.53	144.01	x	x	x
C3	610.09	304.54	202.69	x	x	x
C4	931.07	465.03	309.69	x	x	x
C5	1187.06	593.03	395.02	x	x	x
C6	1508.05	753.52	502.01	x	x	x
Z0	4.12	1.56	0.70	x	x	x
Z1	325.11	162.05	107.70	x	x	x
Z2	581.10	290.04	193.03	x	x	x
Z3	902.08	450.54	300.02	x	x	x
Z4	1078.11	538.55	358.70	x	x	x
Z5	1399.09	699.04	465.69	x	x	x

[1] Columns represent different charge states.

[2] Each CCS is an average of independent measurements with the corresponding standard deviation

Supplementary Table 49. CCS of B, Y, C and Z (-SO_3) ions identified in a hexasaccharide G-GlcNS6S-G-GlcNS3S-I2S-GlcNS6S-R_1 structure, where R_1 is $(CH_2)_5NH_2$.

**#34**						
G-GlcNS6S-G-GlcNS3S-I2S-GlcNS6S-R_1						
	- $1SO_3$	- $1SO_3$	- $1SO_3$	- $1SO_3$	- $1SO_3$	- $1SO_3$
	(1-)[1]	(2-)	(3-)	(1-)	(2-)	(3-)
B1	95.07	47.03	31.02	x	x	x
B2	416.05	207.52	138.01	x	121.8 (0.4)[2]	x
B3	592.08	295.54	196.69	139.1 (0.3)	X	x
B4	913.06	456.03	303.68	x	x	x
B5	1169.05	584.02	389.01	x	x	x
B6	1490.04	744.51	496.01	x	x	x
Y0	22.14	10.56	6.71	x	x	x
Y1	343.12	171.05	113.70	109.5 (0.3)	x	x
Y2	599.11	299.05	199.03	x	156.4 (0.1)	x
Y3	920.09	459.54	306.02	x	x	x
Y4	1096.12	547.56	364.70	x	209.9 (0.5)	x
Y5	1417.10	708.05	471.70	x	x	x
C1	113.08	56.04	37.02	x	x	x
C2	434.06	216.53	144.01	x	x	x
C3	610.09	304.54	202.69	x	x	x
C4	931.07	465.03	309.69	x	x	x
C5	1187.06	593.03	395.02	x	x	x
C6	1508.05	753.52	502.01	x	x	x
Z0	4.12	1.56	0.70	x	x	x
Z1	325.11	162.05	107.70	x	x	x
Z2	581.10	290.04	193.03	x	x	x
Z3	902.08	450.54	300.02	x	x	x
Z4	1078.11	538.55	358.70	x	x	x
Z5	1399.09	699.04	465.69	x	x	x

[1] Columns represent different charge states.

[2] Each CCS is an average of independent measurements with the corresponding standard deviation

Supplementary Table 50. CCS of B, Y, C and Z (-SO_3) ions identified in a hexasaccharide G-GlcNS6S-G-GlcNS6S3S-I2S-GlcNS6S-R_1 structure, where R_1 is $(CH_2)_5NH_2$.

**#35**						
G-GlcNS6S-G-GlcNS6S3S-I2S-GlcNS6S-R_1						
	- $1SO_3$	- $1SO_3$	- $1SO_3$	- $1SO_3$	- $1SO_3$	- $1SO_3$
	(1-)[1]	(2-)	(3-)	(1-)	(2-)	(3-)
B1	95.07	47.03	31.02	x	x	x
B2	416.05	207.52	138.01	x	122.4 (0.2)	x
B3	592.08	295.54	196.69	139.3 (0.3)	x	x
B4	993.02	496.01	330.34	x	x	x
B5	1249.01	624.00	415.66	x	x	x
B6	1569.99	784.49	522.66	x	x	x
Y0	22.14	10.56	6.71	x	x	x
Y1	343.12	171.05	113.70	109.8 (0.2)	x	x
Y2	599.11	299.05	199.03	x	x	x
Y3	1000.05	499.52	332.68	x	x	x
Y4	1176.08	587.53	391.35	x	x	242.3 (0.5)
Y5	1497.06	748.03	498.35	x	x	x
C1	113.08	56.04	37.02	x	x	x
C2	434.06	216.53	144.01	x	x	x
C3	610.09	304.54	202.69	x	x	x
C4	1011.03	505.01	336.34	x	x	x
C5	1267.02	633.01	421.67	x	x	x
C6	1588.00	793.50	528.66	x	x	x
Z0	4.12	1.56	0.70	x	x	x
Z1	325.11	162.05	107.70	x	x	x
Z2	581.10	290.04	193.03	x	x	x
Z3	982.03	490.51	326.67	x	x	x
Z4	1158.07	578.53	385.35	x	x	x
Z5	1479.05	739.02	492.34	x	x	x

[1] Columns represent different charge states.

[2] Each CCS is an average of independent measurements with the corresponding standard deviation

Supplementary Table 51. CCS of B, Y, C and Z (-SO_3) ions identified in a septa-saccharide G-GlcNS-G-GlcNS-I2S-GlcNS-G-GlcNS-G-R_3 structure.

**#36**						
G-GlcNS-G-GlcNS-I2S-GlcNS-G-GlcNS-G-R_3						
	- 1SO_3	- 1SO_3	- 1SO_3	- 1SO_3	- 1SO_3	- 1SO_3
	(1-)[1]	(2-)	(3-)	(1-)	(2-)	(3-)
B1	77.06	38.02	25.01	x	x	x
B2	318.08	158.54	105.36	x	x	x
B3	574.07	286.53	190.69	x	x	x
B4	815.10	407.04	271.03	x	x	x
B5	991.13	495.06	329.70	x	x	x
B6	1232.15	615.57	410.05	x	x	x
B7	1408.19	703.59	468.72	x	x	x
B8	1546.21	772.60	514.73	x	x	x
Y0	58.06	28.53	18.68	x	x	x
Y1	234.09	116.54	77.36	x	x	x
Y2	475.12	237.06	157.70	x	x	x
Y3	651.15	325.07	216.38	x	x	x
Y4	892.18	445.58	296.72	x	x	x
Y5	1148.17	573.58	382.05	x	x	x
Y6	1389.19	694.09	462.39	x	x	x
Y7	1547.21	773.10	515.07	x	x	x
C1	94.06	46.53	30.68	x	x	x
C2	335.09	167.04	111.02	x	x	x
C3	591.07	295.03	196.35	x	x	x
C4	832.10	415.55	276.69	x	x	x
C5	1008.13	503.56	335.37	x	x	x
C6	1249.16	624.07	415.71	x	x	x
C7	1425.19	712.09	474.39	x	x	x
C8	1563.21	781.10	520.40	x	x	x
Z0	40.05	19.52	12.68	x	x	x
Z1	216.08	107.54	71.36	x	x	x
Z2	457.11	228.05	151.70	x	x	x
Z3	633.14	316.07	210.38	x	x	x
Z4	874.17	436.58	290.72	x	x	x
Z5	1130.16	564.57	376.05	x	x	x
Z6	1371.18	685.09	456.39	x	x	x
Z7	1546.21	772.60	514.73	x	x	x

[1] Columns represent different charge states.

[2] Each CCS is an average of independent measurements with the corresponding standard deviation

Supplementary Table 52. Summary of B, Y, C and Z ions theoretically identified from one of the six possible hexasaccharide sequences (dp6 + $3SO_3$ + 1NAc) as a result of one glycosidic bond cleavage in the structure ΔUA-GlcNS-UA-GlcNAc-UA2S-GlcNS.

ΔUA-GlcNS-UA-GlcNAc-UA2S-GlcNS				
	(1-)	(2-)	(1-)	(2-)
B1	157.014	78.003	✓	x
B2	398.039	198.516	x	✓
B3	574.071	286.532	✓	✓
B4	777.151	388.071	x	x
B5	1033.140	516.066	x	x
Y0	258.028	128.510	x	x
Y1	514.017	256.505	x	x
Y2	717.097	358.044	x	x
Y3	893.129	446.060	x	x
Y4	1134.154	566.573	x	x
C1	175.024	87.008	✓	x
C2	416.050	207.521	✓	✓
C3	592.082	295.537	✓	✓
C4	795.161	397.077	x	x
C5	1051.150	525.071	x	x
Z0	240.018	119.505	✓	x
Z1	496.007	247.499	x	✓
Z2	699.086	349.039	x	x
Z3	875.118	437.055	x	x
Z4	1116.144	557.568	x	x
			Total	8

Supplementary Table 53. Summary of B, Y, C and Z ions theoretically identified from one of the six possible hexasaccharide sequences (dp6 + $3SO_3$ + 1NAc) as a result of one glycosidic bond cleavage in the structure ΔUA-GlcNS-UA2S-GlcNS-UA-GlcNAc.

ΔUA-GlcNS-UA2S-GlcNS-UA-GlcNAc				
	(1-)	(2-)	(1-)	(2-)
B1	157.014	78.003	✓	x
B2	398.039	198.516	✓	✓
B3	654.028	326.510	x	✓
B4	895.054	447.023	x	x
B5	1071.086	535.039	x	✓
Y0	220.082	109.537	x	x
Y1	396.114	197.553	✓	x
Y2	637.140	318.066	✓	x
Y3	893.129	446.060	x	✓
Y4	1134.154	566.573	x	x
C1	175.024	87.008	✓	x
C2	416.050	207.521	✓	✓
C3	672.039	335.515	x	x
C4	913.064	456.028	x	x
C5	1089.096	544.044	x	x
Z0	202.072	100.532	x	x
Z1	378.104	188.548	✓	x
Z2	619.129	309.061	✓	x
Z3	875.118	437.055	x	x
Z4	1116.144	557.568	x	x
			Total	11

Supplementary Table 54. Summary of B, Y, C and Z ions theoretically identified from one of the six possible hexasaccharide sequences (dp6 + 3SO_3 + 1NAc) as a result of one glycosidic bond cleavage in the structure ΔUA-GlcNAc-UA-GlcNS-UA2S-GlcNS

ΔUA-GlcNAc-UA-GlcNS-UA2S-GlcNS				
	(1-)	(2-)	(1-)	(2-)
B1	157.014	78.003	✓	x
B2	360.093	179.543	x	x
B3	536.125	267.559	✓	x
B4	777.151	388.071	x	x
B5	1033.140	516.066	x	x
Y0	258.028	128.510	x	x
Y1	514.017	256.505	x	x
Y2	755.043	377.017	x	x
Y3	931.075	465.034	x	x
Y4	1134.154	566.573	x	x
C1	175.024	87.008	✓	x
C2	378.104	188.548	✓	x
C3	554.136	276.564	x	x
C4	795.161	397.077	x	x
C5	1051.150	525.071	x	x
Z0	240.018	119.505	✓	x
Z1	496.007	247.499	x	✓
Z2	737.032	368.012	x	x
Z3	913.064	456.028	x	x
Z4	1116.144	557.568	x	x
			Total	6

Supplementary Table 55. Summary of B, Y, C and Z ions theoretically identified from one of the six possible hexasaccharide sequences (dp6 + $3SO_3$ + 1NAc) as a result of one glycosidic bond cleavage in the structure ΔUA-GlcNAc-UA2S-GlcNS-UA-GlcNS.

ΔUA-GlcNAc-UA2S-GlcNS-UA-GlcNS				
	(1-)	(2-)	(1-)	(2-)
B1	157.014	78.003	✓	x
B2	360.093	179.543	x	x
B3	616.082	307.537	x	✓
B4	857.107	428.050	x	x
B5	1033.140	516.066	x	x
Y0	258.028	128.510	x	x
Y1	434.060	216.526	x	x
Y2	675.086	337.039	x	x
Y3	931.075	465.034	x	x
Y4	1134.154	566.573	x	x
C1	175.024	87.008	✓	x
C2	378.104	188.548	✓	x
C3	634.092	316.542	x	x
C4	875.118	437.055	x	x
C5	1051.150	525.071	x	x
Z0	240.018	119.505	✓	x
Z1	416.050	207.521	✓	✓
Z2	657.075	328.034	✓	✓
Z3	913.064	456.028	x	x
Z4	1116.144	557.568	x	x
			Total	7

Supplementary Table 56. Summary of B, Y, C and Z ions theoretically identified from one of the six possible hexasaccharide sequences (dp6 + $3SO_3$ + 1NAc) as a result of one glycosidic bond cleavage in the structure ΔUA2S-GlcNS-UA-GlcNS-UA-GlcNAc.

ΔUA2S-GlcNS-UA-GlcNS-UA-GlcNAc				
	(1-)	(2-)	(1-)	(2-)
B1	236.970	117.981	x	x
B2	477.996	238.494	x	x
B3	654.028	326.510	x	✓
B4	895.054	447.023	x	x
B5	1071.086	535.039	x	x
Y0	220.082	109.537	x	x
Y1	396.114	197.553	✓	x
Y2	637.140	318.066	✓	x
Y3	813.172	406.082	x	✓
Y4	1054.197	526.595	x	✓
C1	254.981	126.987	x	x
C2	496.007	247.499	x	✓
C3	672.039	335.515	x	x
C4	913.064	456.028	x	x
C5	1089.096	544.044	x	x
Z0	202.072	100.532	x	x
Z1	378.104	188.548	✓	x
Z2	619.129	309.061	✓	x
Z3	795.161	397.077	x	x
Z4	1036.187	517.590	x	x
			Total	8

Supplementary Table 57. Summary of B, Y, C and Z ions theoretically identified from one of the six possible hexasaccharide sequences (dp6 + $3SO_3$ + 1NAc) as a result of one glycosidic bond cleavage in the structure ΔUA2S-GlcNS-UA-GlcNAc-UA-GlcNS.

ΔUA2S-GlcNS-UA-GlcNAc-UA-GlcNS				
	(1-)	(2-)	(1-)	(2-)
B1	236.970	117.981	x	x
B2	477.996	238.494	x	x
B3	654.028	326.510	x	✓
B4	857.107	428.050	x	x
B5	1033.140	516.066	x	x
Y0	258.028	128.510	x	x
Y1	434.060	216.526	x	x
Y2	637.140	318.066	✓	x
Y3	813.172	406.082	x	✓
Y4	1054.197	526.595	x	✓
C1	254.981	126.987	x	x
C2	496.007	247.499	x	✓
C3	672.039	335.515	x	x
C4	875.118	437.055	x	x
C5	1051.150	525.071	x	x
Z0	240.018	119.505	✓	x
Z1	416.050	207.521	✓	✓
Z2	619.129	309.061	✓	x
Z3	795.161	397.077	x	x
Z4	1036.187	517.590	x	x
			Total	9

Supplementary Table 58. Summary overview of the B, Y, C and Z ions theoretically identified from one glycosidic bond cleavage in the purified hexasaccharide.

Possible structures	B/C/Y/Z ions
ΔUA-GlcNS-UA-GlcNAc-UA2S-GlcNS	8
ΔUA-GlcNS-UA2S-GlcNS-UA-GlcNAc	11
ΔUA-GlcNAc-UA-GlcNS-UA2S-GlcNS	6
ΔUA-GlcNAc-UA2S-GlcNS-UA-GlcNS	7
ΔUA2S-GlcNS-UA-GlcNS-UA-GlcNAc	8
ΔUA2S-GlcNS-UA-GlcNAc-UA-GlcNS	9

Supplementary Table 59. CCS of B, Y, C and Z ions identified in a ΔUA-GlcNS-I2S-GlcNS-G-GlcNAc structure.

**#HS1**				
ΔUA-GlcNS-I2S-GlcNS-G-GlcNAc				
$[M-3H]^{3-}$	430.05	274.0 (0.6)		
	(1-)[1]	(2-)	(1-)	(2-)
B1	157.014	78.003	70.5 (0.9)	x
B2	398.039	198.516	110.1 (0.8)	119.6 (0.4)
B3	654.028	326.510	x	162.0 (0.7)
B4	895.054	447.023	x	201.0 (0.7)
B5	1071.086	535.039	x	x
Y0	220.082	109.537	x	x
Y1	396.114	197.553	116.1 (0.5)	x
Y2	637.140	318.066	151.8 (0.5)	x
Y3	893.129	446.060	x	x
Y4	1134.154	566.573	x	x
C1	175.024	87.008	71.0 (0.6)	x
C2	416.050	207.521	113.6 (0.4)	x
C3	672.039	335.515	x	164.0 (0.8)
C4	913.064	456.028	x	x
C5	1089.096	544.044	x	x
Z0	202.072	100.532	x	x
Z1	378.104	188.548	113.8 (0.6)	x
Z2	619.129	309.061	149.2 (0.2)	x
Z3	875.118	437.055	x	156.2 (0.6)
Z4	1116.144	557.568	x	x

[1] Columns represent different charge states.

[2] Each CCS is an average of independent measurements with the corresponding standard deviation

Supplementary Table 60. CCS of B, Y, C and Z (-SO_3) ions identified in a hexasaccharide ΔUA-GlcNS-I2S-GlcNS-G-GlcNAc structure.

**#HS1**				
ΔUA-GlcNS-I2S-GlcNS-G-GlcNAc				
$[M-2H]^{2-}$	605.6	225.05 (0.4)		
$[M-3H]^{3-}$	403.4	255.39 (0.1)		
	- $1SO_3$	- $1SO_3$	- $1SO_3$	- $1SO_3$
	(1-)[1]	(2-)	(1-)	(2-)
B1	77.06	38.02	x	x
B2	318.08	158.54	x	x
B3	574.07	286.53	x	150.3 (0.8)[2]
B4	815.10	407.04	x	x
B5	991.13	495.06	x	200.1 (0.6)
Y0	140.13	69.56	x	x
Y1	316.16	157.57	x	x
Y2	557.18	278.09	x	x
Y3	813.17	406.08	x	181.47 (0.5)
Y4	1054.20	526.59	x	x
C1	95.07	47.03	x	x
C2	336.09	167.54	x	x
C3	592.08	295.54	x	x
C4	833.11	416.05	x	179.5 (0.5)
C5	1009.14	504.07	x	x
Z0	122.12	60.55	x	x
Z1	298.15	148.57	x	x
Z2	539.17	269.08	x	x
Z3	795.16	397.08	x	x
Z4	1036.19	517.59	x	x

[1] Columns represent different charge states.

[2] Each CCS is an average of independent measurements with the corresponding standard deviation

Supplementary Table 61. Summary of B, Y, C and Z ions theoretically identified from one of the six possible hexasaccharide sequences (dp6 + $5SO_3$ + 1NAc) as a result of one glycosidic bond cleavage in the structure ΔUA-GlcNS-UA-GlcNAc6S-UA2S-GlcNS6S.

ΔUA-GlcNS-UA-GlcNAc6S-UA2S-GlcNS6S						
	(1-)	(2-)	(3-)	(1-)	(2-)	(3-)
B1	157.014	78.003	51.666	✓	x	x
B2	398.039	198.516	132.008	✓	✓	x
B3	574.071	286.532	190.685	✓	✓	x
B4	857.107	428.050	285.031	x	x	x
B5	1113.096	556.044	370.360	x	x	x
Y0	337.985	168.489	111.990	x	x	x
Y1	593.974	296.483	197.319	x	x	x
Y2	877.010	438.001	291.665	x	x	✓
Y3	1053.042	526.017	350.342	x	x	✓
Y4	1294.068	646.530	430.684	x	x	✓
C1	175.024	87.008	57.670	✓	x	x
C2	416.050	207.521	138.011	✓	x	x
C3	592.082	295.537	196.689	x	x	x
C4	875.118	437.055	291.034	x	x	x
C5	1131.107	565.050	376.364	x	x	x
Z0	319.974	159.483	105.986	x	x	x
Z1	575.963	287.478	191.316	x	x	x
Z2	858.999	428.996	285.661	x	x	x
Z3	1035.031	517.012	344.339	x	x	x
Z4	1276.057	637.525	424.680	x	x	□
				Total	9	

Supplementary Table 62. Summary of B, Y, C and Z ions theoretically identified from one of the six possible hexasaccharide sequences (dp6 + $5SO_3$ + 1NAc) as a result of one glycosidic bond cleavage in the structure ΔUA-GlcNS-UA2S-GlcNS6S-UA-GlcNAc6S.

ΔUA-GlcNS-UA2S-GlcNS6S-UA-GlcNAc6S						
	(1-)	(2-)	(3-)	(1-)	(2-)	(3-)
B1	157.014	78.003	51.666	✓	x	x
B2	398.039	198.516	132.008	✓	✓	x
B3	654.028	326.510	217.338	✓	✓	x
B4	975.011	487.001	324.332	x	✓	x
B5	1151.043	575.017	383.009	x	x	✓
Y0	300.039	149.516	99.341	✓	x	x
Y1	476.071	237.532	158.018	✓	✓	x
Y2	797.053	398.023	265.013	x	x	✓
Y3	1053.042	526.017	350.342	x	x	✓
Y4	1294.068	646.530	430.684	x	x	✓
C1	175.024	87.008	57.670	✓	x	x
C2	416.050	207.521	138.011	✓	x	x
C3	672.039	335.515	223.341	✓	x	x
C4	993.021	496.007	330.335	x	x	x
C5	1169.053	584.023	389.013	x	✓	x
Z0	282.028	140.510	93.338	✓	x	x
Z1	458.060	228.526	152.015	✓	✓	x
Z2	779.043	389.018	259.009	x	x	✓
Z3	1035.032	517.012	344.339	x	x	x
Z4	1276.057	637.525	424.681	x	x	✓
				Total	18	

Supplementary Table 63. Summary of B, Y, C and Z ions theoretically identified from one of the six possible hexasaccharide sequences (dp6 + $5SO_3$ + 1NAc) as a result of one glycosidic bond cleavage in the structure ΔUA-GlcNAc6S-UA-GlcNS-UA2S-GlcNS6S

ΔUA-GlcNAc6S-UA-GlcNS-UA2S-GlcNS6S						
	(1-)	(2-)	(3-)	(1-)	(2-)	(3-)
B1	157.014	78.003	51.666	✓	x	x
B2	440.050	219.521	146.011	x	x	x
B3	616.082	307.537	204.689	x	✓	x
B4	857.107	428.050	285.031	x	✓	x
B5	1113.096	556.044	370.360	x	x	x
Y0	337.985	168.489	111.990	x	x	x
Y1	593.974	296.483	197.319	x	x	x
Y2	835.000	416.996	277.661	x	x	x
Y3	1011.032	505.012	336.339	x	x	x
Y4	1294.068	646.530	430.684	x	x	x
C1	175.024	87.008	57.670	✓	x	x
C2	458.060	228.526	152.015	✓	✓	x
C3	634.092	316.542	210.692	x	x	x
C4	875.118	437.055	291.034	x	x	x
C5	1131.107	565.050	376.364	x	x	x
Z0	319.975	159.483	105.986	x	x	x
Z1	575.963	287.478	191.316	x	x	x
Z2	816.989	407.991	271.658	x	x	x
Z3	993.021	496.007	330.335	x	x	x
Z4	1276.057	637.525	424.681	x	x	x
				Total	5	

Supplementary Table 64. Summary of B, Y, C and Z ions theoretically identified from one of the six possible hexasaccharide sequences (dp6 + $5SO_3$ + 1NAc) as a result of one glycosidic bond cleavage in the structure ΔUA-GlcNAc6S-UA2S-GlcNS6S-UA-GlcNS

ΔUA-GlcNAc6S-UA2S-GlcNS6S-UA-GlcNS						
	(1-)	(2-)	(3-)	(1-)	(2-)	(3-)
B1	157.014	78.003	51.666	✓	x	x
B2	440.050	219.521	146.011	x	x	x
B3	696.039	347.515	231.341	x	x	x
B4	1017.021	508.007	338.335	x	x	x
B5	1193.053	596.023	397.013	x	x	x
Y0	258.028	128.510	85.338	x	x	x
Y1	434.060	216.526	144.015	✓	✓	x
Y2	755.043	377.018	251.009	x	x	x
Y3	1011.032	505.012	336.339	x	x	x
Y4	1294.068	646.530	430.684	x	x	x
C1	175.024	87.008	57.670	✓	x	x
C2	458.060	228.526	152.015	✓	✓	x
C3	714.049	356.521	237.345	x	x	x
C4	1035.032	517.012	344.339	x	✓	x
C5	1211.064	605.028	403.016	x	x	x
Z0	240.018	119.505	79.334	✓	x	x
Z1	416.050	207.521	138.011	✓	✓	x
Z2	737.032	368.012	245.006	x	x	✓
Z3	993.021	496.007	330.335	x	x	x
Z4	1276.057	637.525	424.681	x	x	x
				Total	8	

Supplementary Table 65. Summary of B, Y, C and Z ions theoretically identified from one of the six possible hexasaccharide sequences (dp6 + $5SO_3$ + 1NAc) as a result of one glycosidic bond cleavage in the structure ΔUA2S-GlcNS6S-UA-GlcNS-UA-GlcNAc6S

ΔUA2S-GlcNS6S-UA-GlcNS-UA-GlcNAc6S						
	(1-)	(2-)	(3-)	(1-)	(2-)	(3-)
B1	236.970	117.981	78.318	x	x	x
B2	557.953	278.473	185.312	x	x	x
B3	733.985	366.489	243.990	x	x	x
B4	975.011	487.001	324.332	x	x	x
B5	1151.043	575.017	383.009	x	✓	x
Y0	300.039	149.516	99.341	✓	x	x
Y1	476.071	237.532	158.018	✓	✓	x
Y2	717.097	358.044	238.360	✓	✓	x
Y3	893.129	446.060	297.038	x	✓	x
Y4	1214.111	606.552	404.032	x	x	x
C1	254.981	126.987	84.322	✓	x	x
C2	575.963	287.478	191.316	x	x	x
C3	751.996	375.494	249.993	x	x	x
C4	993.021	496.007	330.335	x	✓	x
C5	1169.053	584.023	389.013	x	✓	✓
Z0	282.028	140.510	93.338	✓	x	x
Z1	458.060	228.526	152.015	✓	✓	x
Z2	699.086	349.039	232.357	x	✓	x
Z3	875.118	437.055	291.034	x	x	x
Z4	1196.101	597.546	398.028	x	x	x
				Total	11	

Supplementary Table 66. Summary of B, Y, C and Z ions theoretically identified from one of the six possible hexasaccharide sequences (dp6 + $5SO_3$ + 1NAc) as a result of one glycosidic bond cleavage in the structure ΔUA2S-GlcNS6S-UA-GlcNAc6S-UA-GlcNS

ΔUA2S-GlcNS6S-UA-GlcNAc6S-UA-GlcNS						
	(1-)	(2-)	(3-)	(1-)	(2-)	(3-)
B1	236.970	117.981	78.318	x	x	x
B2	557.953	278.473	185.312	x	x	x
B3	733.985	366.489	243.990	x	x	x
B4	1017.021	508.007	338.335	x	x	x
B5	1193.053	596.023	397.013	x	x	x
Y0	258.028	128.510	85.338	x	x	x
Y1	434.060	216.526	144.015	✓	✓	x
Y2	717.097	358.044	238.360	✓	✓	x
Y3	893.129	446.060	297.038	x	✓	x
Y4	1214.111	606.552	404.032	x	x	x
C1	254.981	126.987	84.322	x	x	x
C2	575.963	287.478	191.316	x	x	x
C3	751.996	375.494	249.993	x	x	x
C4	1035.032	517.012	344.339	x	✓	x
C5	1211.064	605.028	403.016	x	x	x
Z0	240.018	119.505	79.334	✓	x	x
Z1	416.050	207.521	138.011	✓	✓	x
Z2	699.086	349.039	232.357	x	✓	x
Z3	875.118	437.055	291.034	x	x	x
Z4	1196.101	597.546	398.028	x	x	x
				Total	7	

Supplementary Table 67. Summary overview of the B, Y, C and Z ions theoretically identified from one glycosidic bond cleavage in the purified oligosaccharide.

Possible structures	B/Y/C/Z ions
ΔUA-GlcNS-UA-GlcNAc6S-UA2S-GlcNS6S	9
ΔUA-GlcNS-UA2S-GlcNS6S-UA-GlcNAc6S	18
ΔUA-GlcNAc6S-UA-GlcNS-UA2S-GlcNS6S	5
ΔUA-GlcNAc6S-UA2S-GlcNS6S-UA-GlcNS	8
ΔUA2S-GlcNS6S-UA-GlcNS-UA-GlcNAc6S	11
ΔUA2S-GlcNS6S-UA-GlcNAc6S-UA-GlcNS	7

Supplementary Table 68. CCS of B, Y, C and Z ions identified in a ΔUA-GlcNS-I2S-GlcNS6S-UA-GlcNAc6S structure.

**#HS2**						
ΔUA-GlcNS-I2S-GlcNS6S-G-GlcNAc6S						
$[M-2H]^{2-}$	362.26	331.0 (0.2)[2]				
$[M-3H]^{3-}$	483.35	265.7 (0.4)				
	(1-)[1]	(2-)	(3-)	(1-)	(2-)	(3-)
B1	157.014	78.003	51.666	70.4 (0.8)	x	x
B2	398.039	198.516	132.008	110.3 (0.5)	x	x
B3	654.028	326.510	217.338	x	162.4 (0.2)	x
B4	975.011	487.001	324.332	x	225.3 (0.5)	x
B5	1151.043	575.017	383.009	x	x	272.9 (0.4)
Y0	300.039	149.516	99.341	94.7 (0.3)	x	x
Y1	476.071	237.532	158.018	123.2 (0.5)	134.1 (0.5)	x
Y2	797.053	398.023	265.013	x	x	199.6 (0.6)
Y3	1053.042	526.017	350.342	x	x	250.4 (0.5)
Y4	1294.068	646.530	430.684	x	x	276.9 (0.4)
C1	175.024	87.008	57.670	70.4 (0.6)	x	x
C2	416.050	207.521	138.011	x	x	x
C3	672.039	335.515	223.341	146.9 (0.7)	x	x
C4	993.021	496.007	330.335	x	x	x
C5	1169.053	584.023	389.013	x	x	x
Z0	282.028	140.510	93.338	93.2 (0.5)	x	x
Z1	458.060	228.526	152.015	120.5 (0.3)	x	x
Z2	779.043	389.018	259.009	X	x	x
Z3	1035.032	517.012	344.339	x	x	x
Z4	1276.057	637.525	424.681	x	x	x

[1] Columns represent different charge states.

[2] Each CCS is an average of independent measurements with the corresponding standard deviation

Supplementary Table 69. CCS of B, Y, C and Z (-SO_3) ions identified in a hexasaccharide ΔUA-GlcNS-I2S-GlcNS6S-G-GlcNAc6S structure.

**#HS2**				
ΔUA-GlcNS-I2S-GlcNS6S-G-GlcNAc6S				
$[M-3H]^{3-}$	342.27	318.8 (0.5)		
	- 1SO_3	- 1SO_3	- 1SO_3	- 1SO_3
	(1-)[1]	(2-)	(1-)	(2-)
B1	77.06	38.02	x	x
B2	318.08	158.54	x	x
B3	574.07	286.53	x	x
B4	895.05	447.02	x	x
B5	1071.09	535.04	x	x
Y0	220.08	109.54	x	x
Y1	396.11	197.55	x	x
Y2	717.10	358.04	x	x
Y3	973.09	486.04	x	x
Y4	1214.11	606.55	x	x
C1	95.07	47.03	x	x
C2	336.09	167.54	x	x
C3	592.08	295.54	145.7 (0.6)[2]	x
C4	913.06	456.03	x	x
C5	1089.10	544.04	x	x
Z0	202.07	100.53	x	x
Z1	378.10	188.55	x	x
Z2	699.09	349.04	x	x
Z3	955.08	477.03	x	x
Z4	1196.10	597.55	x	x

[1] Columns represent different charge states.

[2] Each CCS is an average of independent measurements with the corresponding standard deviation
