# Supplementary material for: Shotgun ion mobility mass spectrometry sequencing of heparan sulfate saccharides
Source: Nat Commun. 2020 Mar 20;11:1481. doi: 10.1038/s41467-020-15284-y (PMC7083916; doi:10.1038/s41467-020-15284-y)
Supplement: Supplementary file 3 — Reporting Summary [file 41467_2020_15284_MOESM3_ESM.pdf]

nature research

Corresponding author(s): Rebecca Miller

Last updated by author(s): Feb 25, 2020

Reporting Summary

Nature Research wishes to improve the reproducibility of the work that we publish. This form provides structure for consistency and transparency in reporting. For further information on Nature Research policies, see Authors & Referees and the Editorial Policy Checklist .

Statistics

For all statistical analyses, confirm that the following items are present in the figure legend, table legend, main text, or Methods section.

n/a	Confirmed	
☐	☒	The exact sample size (*n*) for each experimental group/condition, given as a discrete number and unit of measurement
☐	☒	A statement on whether measurements were taken from distinct samples or whether the same sample was measured repeatedly
☒	☐	The statistical test(s) used AND whether they are one- or two-sided<br>*Only common tests should be described solely by name; describe more complex techniques in the Methods section.*
☒	☐	A description of all covariates tested
☒	☐	A description of any assumptions or corrections, such as tests of normality and adjustment for multiple comparisons
☐	☒	A full description of the statistical parameters including central tendency (e.g. means) or other basic estimates (e.g. regression coefficient) AND variation (e.g. standard deviation) or associated estimates of uncertainty (e.g. confidence intervals)
☒	☐	For null hypothesis testing, the test statistic (e.g. *F*, *t*, *r*) with confidence intervals, effect sizes, degrees of freedom and *P* value noted<br>*Give P values as exact values whenever suitable.*
☒	☐	For Bayesian analysis, information on the choice of priors and Markov chain Monte Carlo settings
☒	☐	For hierarchical and complex designs, identification of the appropriate level for tests and full reporting of outcomes
☒	☐	Estimates of effect sizes (e.g. Cohen's *d*, Pearson's *r*), indicating how they were calculated

Our web collection on statistics for biologists contains articles on many of the points above.

Software and code

Policy information about availability of computer code

Data collection	Mass Lynx V4.1 software (Waters) is used to operate the instrument and save data files.
Data analysis	The algorithm for processing ion-mobility data (Aprid) was written in-house and used for CCS calculation in an automated fashion. It is implemented using Python 2.7 and can be operated by the user through a graphic interface devised in PyQtDesigner. The main goal of the script is to determine collision cross-sections (CCSs) for specific mass-to-charge (m/z) ratios. For that purpose, the script follows the mathematical approach of solving the Mason-Schamp equation. Details about the code and modules can be found online via github: https://github.com/waschbaerlauch/CCS-Calculation

For manuscripts utilizing custom algorithms or software that are central to the research but not yet described in published literature, software must be made available to editors/reviewers. We strongly encourage code deposition in a community repository (e.g. GitHub). See the Nature Research guidelines for submitting code & software for further information.

Data

Policy information about availability of data

All manuscripts must include a data availability statement. This statement should provide the following information, where applicable:

- Accession codes, unique identifiers, or web links for publicly available datasets
- A list of figures that have associated raw data
- A description of any restrictions on data availability

All data generated or analyzed during this study are included in this article and/or associated Supplementary Information tables 1-69 and uploaded to UniCarbDR https://unicarb-dr.biomedicine.gu.se/references/388. The source data underlying Figures 4b and 5d and Supplementary Figure 3b are provided as a Source Data file.

Field-specific reporting

Please select the one below that is the best fit for your research. If you are not sure, read the appropriate sections before making your selection.

- [x] Life sciences
- [ ] Behavioural & social sciences
- [ ] Ecological, evolutionary & environmental sciences

For a reference copy of the document with all sections, see nature.com/documents/nr-reporting-summary-flat.pdf

Life sciences study design

All studies must disclose on these points even when the disclosure is negative.

Sample size	Standard statistical calculations of standard deviation were made using n=3 within individual experiments for the BaF cells assays. Intra-assay variations technical replicates were small. Sample sizes were chosen based on extensive experience with this assay showing that this was sufficient for obtaining statistically significant results of sufficient power.
Data exclusions	Only data excluded were peaks where a CCS value could not be determine as a result of imprefect peak shapes
Replication	Muliple replicates (2-4) were obtained for each CCS value. Biologically BaF3 FGF assays were preformed with both technical replicates (3) and biological replicates (1-3). All attempts were successful.
Randomization	Randomization of sample measurement was not considered appropriate for the studies undertaken, since samples were not being selected from groups for testing and selection bias was not considered to be an issue.
Blinding	Deliberate blinding was not used; it was not considered appropriate since the structures were not analysed until after the biological assay data was obtained.

Reporting for specific materials, systems and methods

We require information from authors about some types of materials, experimental systems and methods used in many studies. Here, indicate whether each material, system or method listed is relevant to your study. If you are not sure if a list item applies to your research, read the appropriate section before selecting a response.

Materials & experimental systems

n/a	Involved in the study	
x		Antibodies
	x	Eukaryotic cell lines
x		Palaeontology
x		Animals and other organisms
x		Human research participants
x		Clinical data

Methods

n/a	Involved in the study	
x		ChIP-seq
x		Flow cytometry
x		MRI-based neuroimaging

Eukaryotic cell lines

Policy information about cell lines

Cell line source(s)	A murine Ba/F3 cell line transfected with human FGF receptor 1c was a gift from Prof David Ornitz, Washington University, St Louis. Parental (untransfected) Ba/F3 can be purchased from the RIKEN cell bank. The original description of transfection with FGF receptor 1c was https://www.ncbi.nlm.nih.gov/pmc/articles/PMC364088/pdf/molcellb00025-0262.pdf
Authentication	Cell lines were not authenticated by the authors
Mycoplasma contamination	Tested negative for mycoplasma
Commonly misidentified lines (See ICLAC register)	None
